# Genomic and biological characterization of lytic phages infecting *Pseudomonas syringae* associated with almond bacterial blast

**DOI:** 10.1038/s41598-026-47496-5

**Published:** 2026-04-07

**Authors:** Cuong V. Hoang, Jonathan Fan, Hajun Lee, Lauren Bhasin, Dominic Nguyen, Ashley Duran, Ryan Quaal, Suraj Ganiger, Luna Santiago, James E. Adaskaveg, Tawanda E. Maguvu, Florent P. Trouillas, Olakunle I. Olawole

**Affiliations:** 1 Dept. of Microbiology & Plant Pathology, University of California, Riverside, USA; 2 Department of Plant Pathology, University of California, Davis, CA USA; 3 Department of Plant Pathology, Kearney Agricultural Research and Extension Center, Parlier, CA USA

**Keywords:** Bacteriophage biocontrol, Almond bacterial blast, Lytic phages, Host range, Multiplicity of infection, Biofilm disruption, Phage genomics, Biotechnology, Microbiology, Plant sciences

## Abstract

*Pseudomonas* species are Gram-negative bacterial pathogens that affect a wide range of economically important crops, including almond. Increasing resistance of *Pseudomonas syringae* to conventional management strategies highlights the need for alternative disease control options. In this study, we isolated and characterized three lytic bacteriophages, including vB_PsyP_Mobley, vB_PsyP_Plaza, and vB_PsyP_Mission, targeting almond-infecting *Pseudomonas* strains. Host-range analysis across 36 isolates revealed partially overlapping infectivity profiles, with strongest activity against phylogroup 2 (PG2) almond-associated *P. syringae* pv. *syringae* and reduced infectivity against phylogenetically distinct isolates, including PG7 *P. viridiflava* and other crop-associated pathovars. Efficiency-of-plating assays quantitatively supported these host-range patterns. Plaque morphology and transmission electron microscopy demonstrated icosahedral capsids with short, non-contractile tails consistent with tailed dsDNA bacteriophages of the class *Caudoviricetes*, with vB_PsyP_Mission producing halo-associated plaques consistent with predicted extracellular polysaccharide-modifying proteins. Genomic analyses revealed compact (~ 40 kb) genomes lacking integrases, tRNAs, lysogeny-associated genes, and known virulence or antimicrobial resistance determinants. Comparative genomics showed that vB_PsyP_Plaza and vB_PsyP_Mission likely represent novel isolates within previously described species-level groups, whereas vB_PsyP_Mobley is more genomically divergent. Collectively, these results define the phenotypic and genomic characteristics of three related but distinguishable *Pseudomonas* phages and provide a basis for future *in planta* evaluation of their potential utility against almond bacterial blast.

**Supplementary Information:**

The online version contains supplementary material available at 10.1038/s41598-026-47496-5.

## Introduction

*Pseudomonas* species are Gram-negative bacteria with an exceptionally broad host range, which includes fruits, vegetables, cereals, ornamentals, and woody crops worldwide. These pathogens cause blights, cankers, leaf spots, wilts, and soft rots, resulting in substantial losses in global agricultural productivity^1,2^. Among this genus, *Pseudomonas syringae* is widely recognized as one of the most destructive plant pathogens worldwide. It encompasses numerous pathovars that collectively infect more than 180 host species, often causing severe disease outbreaks that can lead to yield losses approaching 80% in susceptible crops^3,4^. The ecological versatility of *P. syringae*, its ability to thrive on leaf surfaces, persist in water systems, and disperse via aerosols or rainfall, contributes to its widespread distribution and recurrent epidemic cycles^5^.

Within this diverse species, almond-infecting *P. syringae* pv. *syringae* (*Pss*) has emerged as a particularly serious threat. Almonds are California’s most valuable specialty crop with a $5 billion market value, and account for over 80% of global production^6^. Bacterial blast, caused by *Pss*, has intensified in recent years, leading to shoot dieback, blossom necrosis, and premature fruit loss, symptoms that are especially damaging to young orchards. Outbreaks are most severe during the cold, wet springs typical of California’s Central Valley^7^. Recent epidemics (2019–2021 and 2023) caused crop losses of up to 40% in affected orchards, highlighting the growing vulnerability of almond production systems^8^.

Current management strategies depend primarily on copper-based bactericides and kasugamycin (Kasumin), the latter of which is used under repeated Sect. 18 emergency exemptions^7^. However, copper resistance is now widespread, Kasumin efficacy is inconsistent, and tightening chemical regulations are reducing the availability of these tools^9,10^. In addition to declining performance, these products accumulate in soil, disrupt beneficial microbiota, and pose environmental and worker-safety concerns^11,12^. Collectively, these challenges highlight an urgent need for biologically based, sustainable alternatives that reduce pesticide reliance and enhance the long-term resilience of specialty crops worldwide.

Bacteriophages (phages) have re-emerged as promising biological control agents for plant pathogens^13,14^. Phage-based interventions offer several advantages: they exhibit high host specificity, minimizing damage to beneficial microbiota; they replicate in proportion to bacterial densities, enabling self-sustaining control; and they evolve alongside their bacterial hosts, potentially reducing the risk of long-term resistance^15,16^. Importantly, phage-based biocontrol aligns with integrated pest management and sustainable agriculture frameworks that prioritize reduced chemical input and the deployment of environmentally compatible disease management tools^13,17^. Previous studies have demonstrated the efficacy of lytic phages in suppressing *P. syringae* populations in vitro and *in planta*, delaying disease progression, and reducing symptom severity across multiple crops^15,18–22^.

Despite these advances, phages targeting almond-associated *P. syringae* pv. *syringae* remain poorly characterized. In this study, we isolated three distinct phages, including vB_PsyP_Mobley, vB_PsyP_Plaza, and vB_PsyP_Mission, from sewage and soil samples and performed an integrated genomic, phylogenomic, and functional characterization to evaluate their potential as biocontrol agents against almond bacterial blast. We examined phage morphology, infection dynamics, environmental stability, and lytic activity to determine whether or not these phages could be viable tools for biocontrol efforts. We then quantified infectivity across a diverse panel of *Pseudomonas* isolates representing multiple crops and ecological backgrounds, including phylogenetically defined host groups (PG2 and PG7). We further conducted comparative genomic analyses to define their genomic features and identify traits relevant to biocontrol, including lytic modules, the absence of lysogeny-associated genes, and variation in predicted host-interaction proteins such as receptor-binding and depolymerase-associated candidates. Collectively, these findings expand current understanding of phage diversity, host specificity, and infection dynamics within the almond *Pseudomonas* pathosystem and provide a foundation for developing phage-based strategies to enhance sustainable disease management in perennial specialty crop systems.

## Results

### Phage selection and morphological characterization

**Fig. 1:**
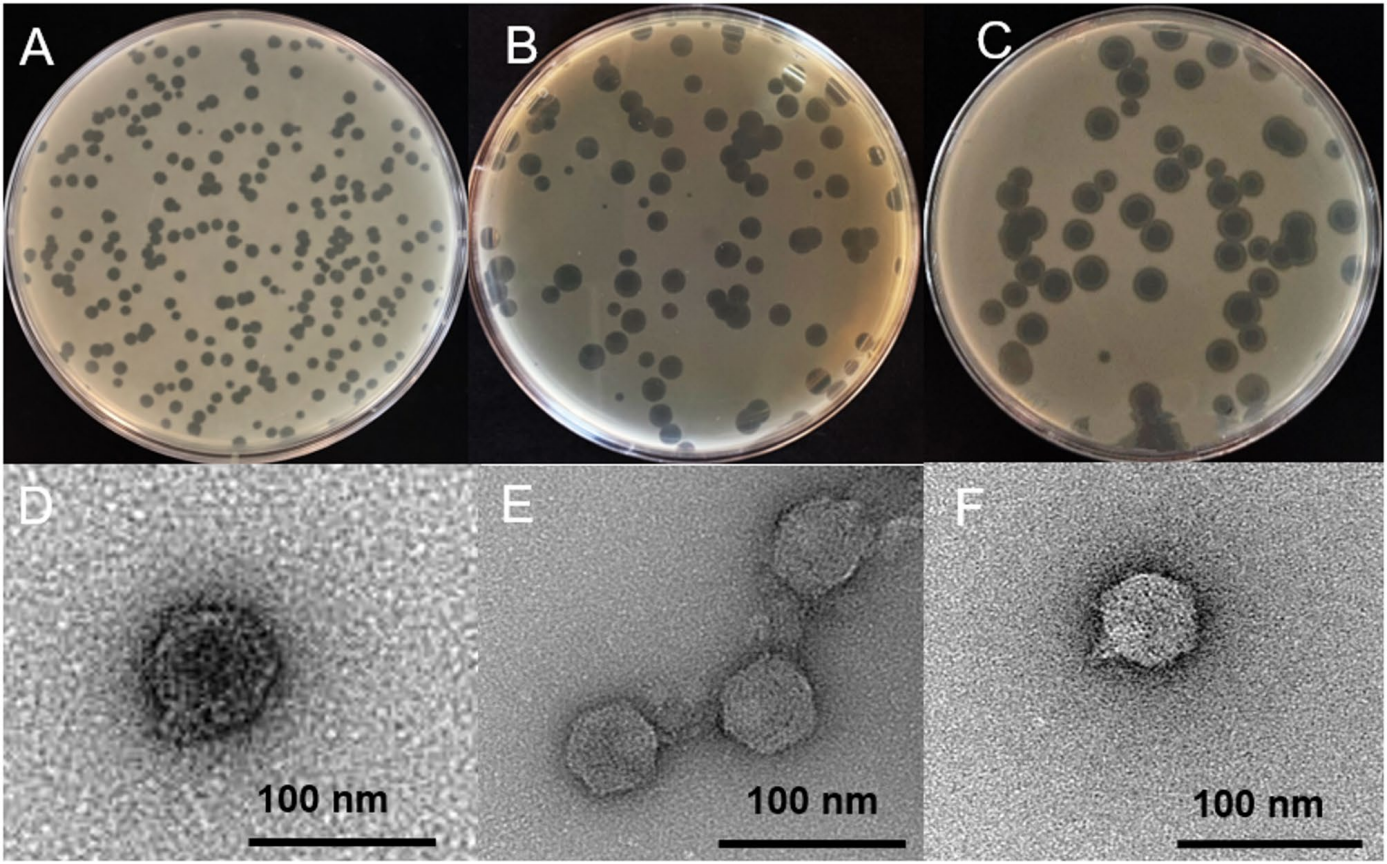
Morphological characteristics of almond-infecting *Pseudomonas* phages propagated on host strain *Pseudomonas syringae* pv. *syringae* 2507. Plaque morphology of vB_PsyP_Mobley (**A**), vB_PsyP_Plaza (**B**), and vB_PsyP_Mission (**C**) following propagation on *Pseudomonas syringae* pv. *syringae* strain 2507. vB_PsyP_Mobley produces small, well-defined circular plaques lacking halos, vB_PsyP_Plaza forms larger, uniformly clear plaques, and vB_PsyP_Mission generates plaques surrounded by distinct turbid halos. Transmission electron micrographs (D–F) show representative virions of vB_PsyP_Mobley (**D**), vB_PsyP_Plaza (**E**), and vB_PsyP_Mission (**F**), each displaying icosahedral capsids and short, non-contractile tails characteristic of tailed dsDNA bacteriophages within the class *Caudoviricetes*. Scale bars are indicated in each panel.

Representative phages were selected for detailed characterization based on distinct restriction digest profiles, with vB_PsyP_Mobley originating from soil-derived filtrates collected near UC Riverside and vB_PsyP_Plaza and vB_PsyP_Mission originating from sewage-derived filtrates. For mnemonic purposes, phages were named after street names within Riverside County, California, and candidate names were verified for uniqueness through literature searches and PhagesDB prior to assignment. All phages were prefixed with vB_PsyP in accordance with ICTV/BAVS nomenclature guidelines, reflecting their isolation and propagation on a *Pseudomonas syringae* host. Plaque assays revealed clear differences in plaque morphology among the three phages (Fig. 1A–C). vB_PsyP_Mobley produced small, well-defined plaques (Fig. 1A). In contrast, vB_PsyP_Plaza formed larger, uniformly clear plaques (Fig. 1B), whereas vB_PsyP_Mission produced large plaques surrounded by distinct halos (Fig. 1C), a phenotype commonly associated with extracellular polysaccharide–modifying activity. Transmission electron microscopy further showed that all three phages possess icosahedral capsids and short tails characteristic of the class *Caudoviricetes* (Fig. 1D–F). Capsid dimensions were comparable across phages, with mean widths of 58.47 ± 4.86 nm for vB_PsyP_Mobley, 53.87 ± 2.34 nm for vB_PsyP_Plaza, and 54.15 ± 1.20 nm for vB_PsyP_Mission (Table 1). Tail lengths differed modestly, with vB_PsyP_Mobley exhibiting the longest mean tail length (14.29 ± 3.64 nm), followed by vB_PsyP_Mission (10.60 ± 0.33 nm) and vB_PsyP_Plaza (9.23 ± 3.12 nm). Consistent with these measurements, all three phages exhibited short, non-contractile tails consistent with podovirus-like morphology.

**Table 1:** General, physiology, and structural characteristics of phages vB_PsyP_Mobley, vB_PsyP_Mission and vB_PsyP_Plaza.

Features	vB_PsyP_Mobley	vB_PsyP_Plaza	vB_PsyP_Mission
Mean capsid width (nm) (SD)^a^	58.47 (±4.86)	53.87 (±2.34)	54.15 (±1.20)
Mean tail length (nm) (SD)^a^	14.29 (±3.64)	9.23 (±3.12)	10.60 (±0.33)

^a^ Phage physical dimensions are the means of measurements of four virions, and values in parentheses indicate standard deviations.

### Host-range and efficiency-of-plating analysis across almond-associated and phylogenetically distinct *Pseudomonas* isolates

**Fig. 2:**
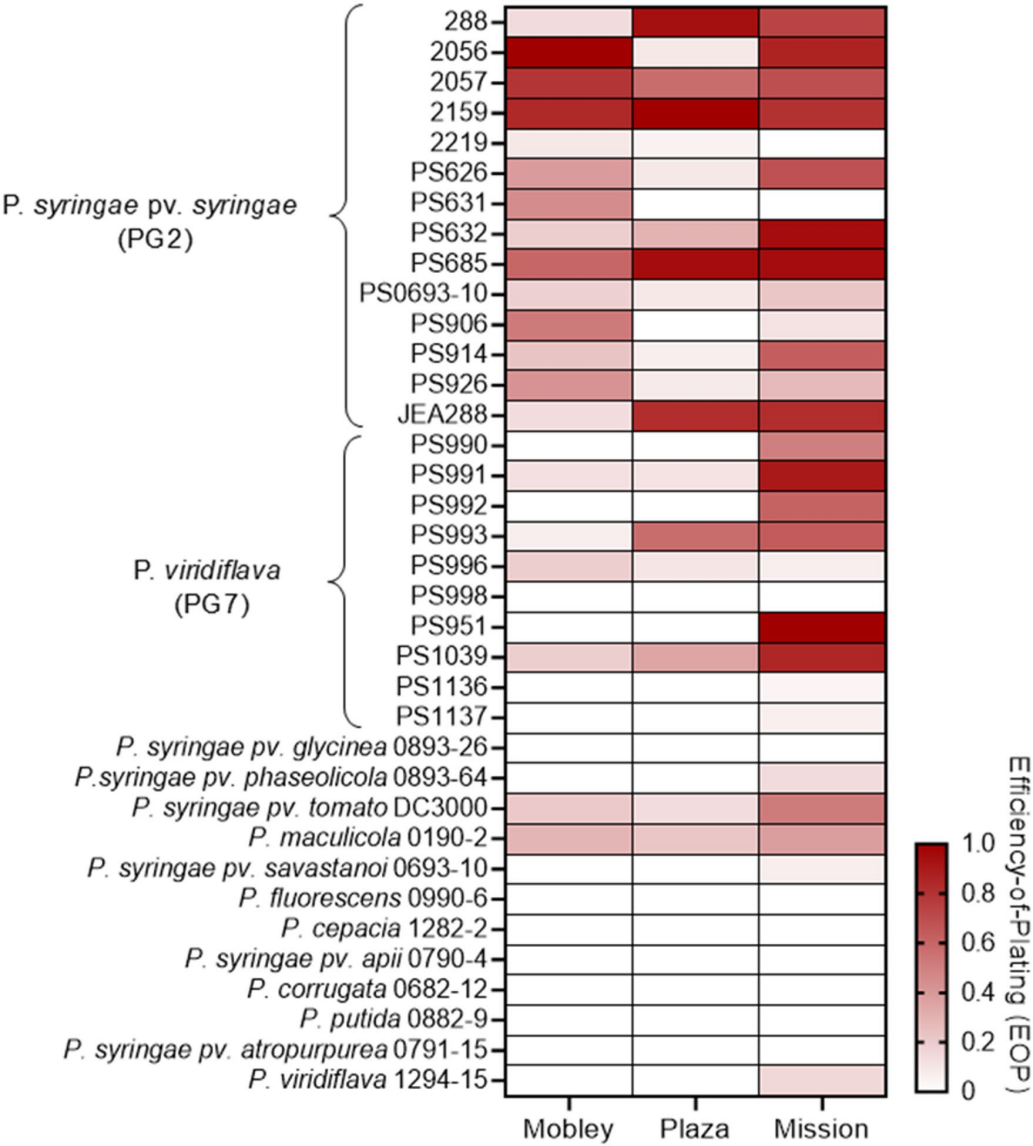
Efficiency-of-plating profiles of three *P. syringae* phages across a diverse *Pseudomonas* panel. Efficiency of plating (EOP) was quantified for vB_PsyP_Mobley, vB_PsyP_Plaza, and vB_PsyP_Mission across the indicated *Pseudomonas* isolates/pathovars. For each phage, plaque counts on each test host were normalized to the host yielding the highest plaque titer (set to 1.0), and EOP values for all other hosts were calculated relative to this maximum. Heatmap shading reflects relative plating efficiency (white = no detectable plaques; darker red = higher EOP). Isolates are grouped by phylogroup/pathovar, highlighting strong plating on PG2 *P. syringae* pv. *syringae* and more variable, generally reduced plating on PG7 *P. viridiflava* and other non-almond–associated *Pseudomonas* taxa.

Host-range and efficiency-of-plating (EOP) analyses using a panel of 36 *Pseudomonas* isolates revealed distinct yet partially overlapping infectivity profiles among the three phages (Fig. 2, Table S1). Almond-associated *Pseudomonas syringae* pv. *syringae* isolates included in this study were previously recovered from symptomatic almond tissues and extensively characterized, providing a biologically relevant host set^7^. These isolates belong primarily to phylogroup 2 (PG2), with additional representation from the phylogenetically distinct phylogroup 7 (PG7), enabling assessment of phage activity across related but divergent lineages. All three phages produced clear plaques and generally high EOP values across the majority of PG2 isolates, indicating efficient productive infection within this clade. In contrast, infectivity against PG7 *P. viridiflava* isolates was more variable and typically associated with lower EOP values, reflecting reduced infection efficiency. Among the three phages, vB_PsyP_Mission exhibited the broadest and most consistent activity across PG7 isolates and also infected several non-almond *Pseudomonas* pathovars, including strains associated with bean (*pv. phaseolicola*), tomato (*pv. tomato* DC3000), cauliflower (*P. maculicola*), oleander (*pv. savastanoi*), and radish (*P. viridiflava*), albeit at lower efficiencies. In contrast, vB_PsyP_Mobley and vB_PsyP_Plaza infected a narrower subset of non-almond isolates, primarily tomato- and cauliflower-associated strains, with both plaque formation and EOP values generally lower than those observed on almond-associated PG2 hosts. No productive infection (EOP ≈ 0) was detected for any phage against more phylogenetically distant *Pseudomonas* species, including *P. syringae* pv. *glycinea*, *P. fluorescens*, *P. cepacia*, *P. corrugata*, *P. putida*, *P. syringae* pv. *apii*, or *P. syringae* pv. *atropurpurea*. Collectively, these results demonstrate strong adaptation to PG2 almond-associated *P. syringae* pv. *syringae*, with reduced and often partial infectivity against phylogenetically distinct PG7 *P. viridiflava* and other non-almond *Pseudomonas* pathovars, consistent with clade-associated patterns of phage–host interaction.

### Optimal multiplicity of infection

The optimal multiplicity of infection (MOI) for each phage was determined by infecting cultures of the almond-infecting *Pseudomonas syringae* pv. *syringae* strain 2057 with vB_PsyP_Mobley, vB_PsyP_Mission, and vB_PsyP_Plaza across a range of MOIs and monitoring phage titers over time. vB_PsyP_Mobley and vB_PsyP_Plaza exhibited maximal replication efficiency at an MOI of 10^− 3^ (Tables 2 and 3), whereas vB_PsyP_Mission reached peak propagation at an MOI of 10^− 4^ (Table 4). These MOI values corresponded to the conditions yielding the highest phage titers while maintaining effective bacterial suppression during the assay period.

**Table 2:** Determination of optimal multiplicity of infection (MOI) of phage vB_PsyP_Mobley.

MOI	Initial Concentration		Final concentration
	Bacterial host (CFU/mL)	Phage (PFU/mL)	Phage (PFU/mL)^1^
10	4.55 × 10^8^	1.7 × 10^10^	(3.03 ± 0.15) x 10^8^
1	4.55 × 10^8^	1.7 × 10^9^	(2.23 ± 1.32) x 10^8^
10^−1^	4.55 × 10^8^	1.7 × 10^8^	(6.97 ± 6.23) x 10^8^
10^−2^	4.55 × 10^8^	1.7 × 10^7^	(1.23 ± 0.23) x 10^9^
10^−3^	4.55 × 10^8^	1.7 × 10^6^	(1.62 ± 0.37) x 10^9^
10^−4^	4.55 × 10^8^	1.7 × 10^5^	(1.41 ± 0.40) x 10^9^

^1^Final concentrations are reported as mean ± SD from three independent replicates.

**Table 3:** Determination of optimal multiplicity of infection (MOI) of phage vB_PsyP_Plaza.

MOI	Initial Concentration		Final concentration
	Bacterial host (CFU/mL)	Phage (PFU/mL)	Phage (PFU/mL)^1^
10	4.55 × 10^8^	4.7 × 10^10^	(7.2 ± 0.33) x 10^8^
1	4.55 × 10^8^	4.7 × 10^9^	(7.57 ± 0.64) x 10^8^
10^−1^	4.55 × 10^8^	4.7 × 10^8^	(7.07 ± 0.27) x 10^8^
10^−2^	4.55 × 10^8^	4.7 × 10^7^	(1.06 ± 0.15) x 10^9^
10^−3^	4.55 × 10^8^	4.7 × 10^6^	(2.05 ± 0.50) x 10^9^
10^−4^	4.55 × 10^8^	4.7 × 10^5^	(1.44 ± 0.14) x 10^9^

^1^Final concentrations are reported as mean ± SD from three independent replicates.

**Table 4:** Determination of optimal multiplicity of infection (MOI) of phage vB_PsyP_Mission.

MOI	Initial Concentration		Final concentration
	Bacterial host (CFU/mL)	Phage (PFU/mL)	Phage (PFU/mL)^1^
10	4.55 × 10^8^	2.5 × 10^10^	(1.87 ± 0.39) x 10^9^
1	4.55 × 10^8^	2.5 × 10^9^	(1.84 ± 0.14) x 10^9^
10^−1^	4.55 × 10^8^	2.5 × 10^8^	(2.13 ± 0.12) x 10^9^
10^−2^	4.55 × 10^8^	2.5 × 10^7^	(2.19 ± 0.28) x 10^9^
10^−3^	4.55 × 10^8^	2.5 × 10^6^	(1.42 ± 0.15) x 10^9^
10^−4^	4.55 × 10^8^	2.5 × 10^5^	(2.24 ± 0.06) x 10^9^

^1^Final concentrations are reported as mean ± SD from three independent replicates.

### Lytic activity against planktonic and biofilm cells

**Fig. 3:**
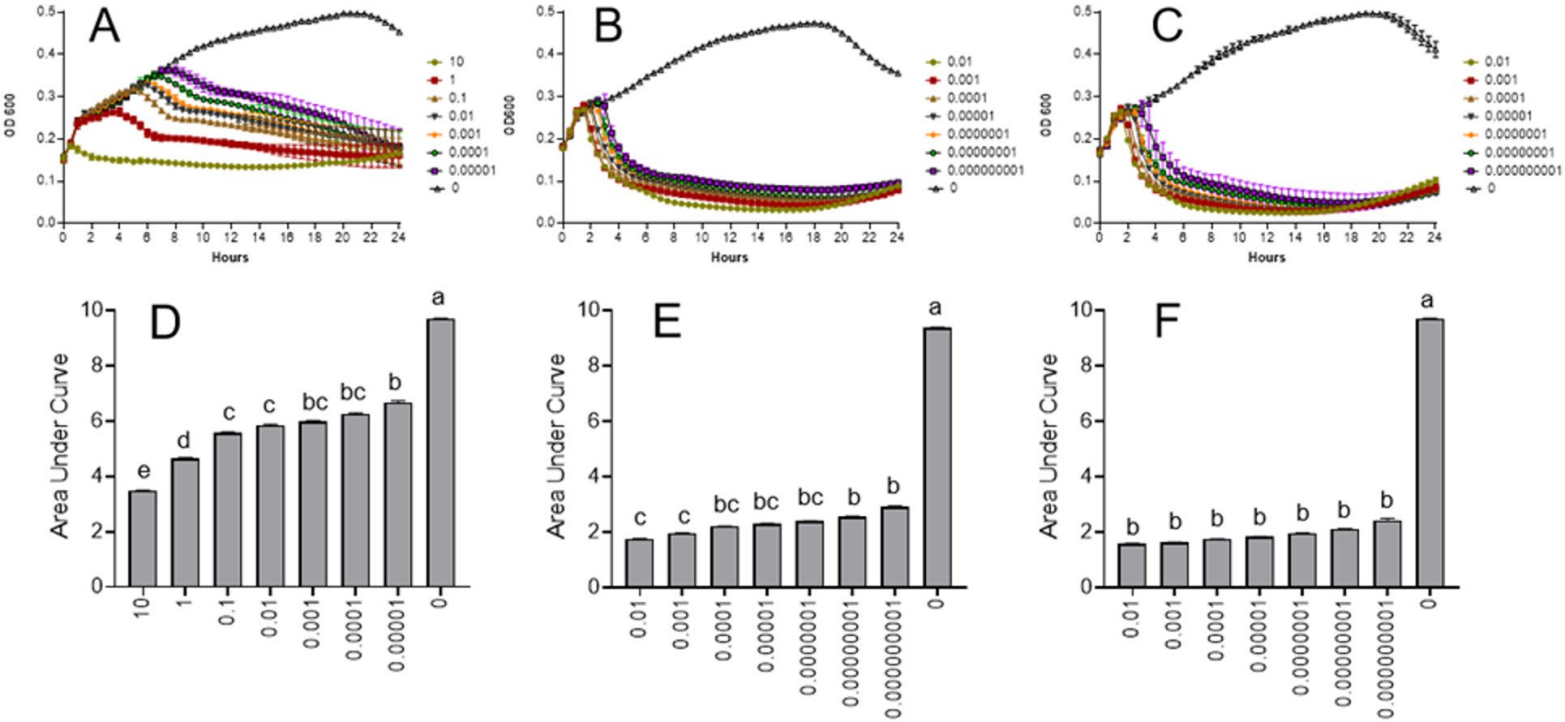
Lytic activity of phages against *Pseudomonas* planktonic cells. Bacterial growth curves (OD₆₀₀) of *Pseudomonas syringae* pv. *syringae* 2507 treated with phages vB_PsyP_Mobley (**A**), vB_PsyP_Plaza (**B**), and vB_PsyP_Mission (**C**) at the indicated multiplicities of infection (MOIs). Corresponding areas under the growth curves (AUC) are shown in panels (**D–F**), respectively. Data represent the mean ± SD of three technical replicates from at least two independent experiments. Statistical differences among treatments were assessed using one-way ANOVA followed by Tukey’s multiple comparisons test. Bars sharing the same lowercase letter are not significantly different (*p* < 0.05).

**Fig. 4:**
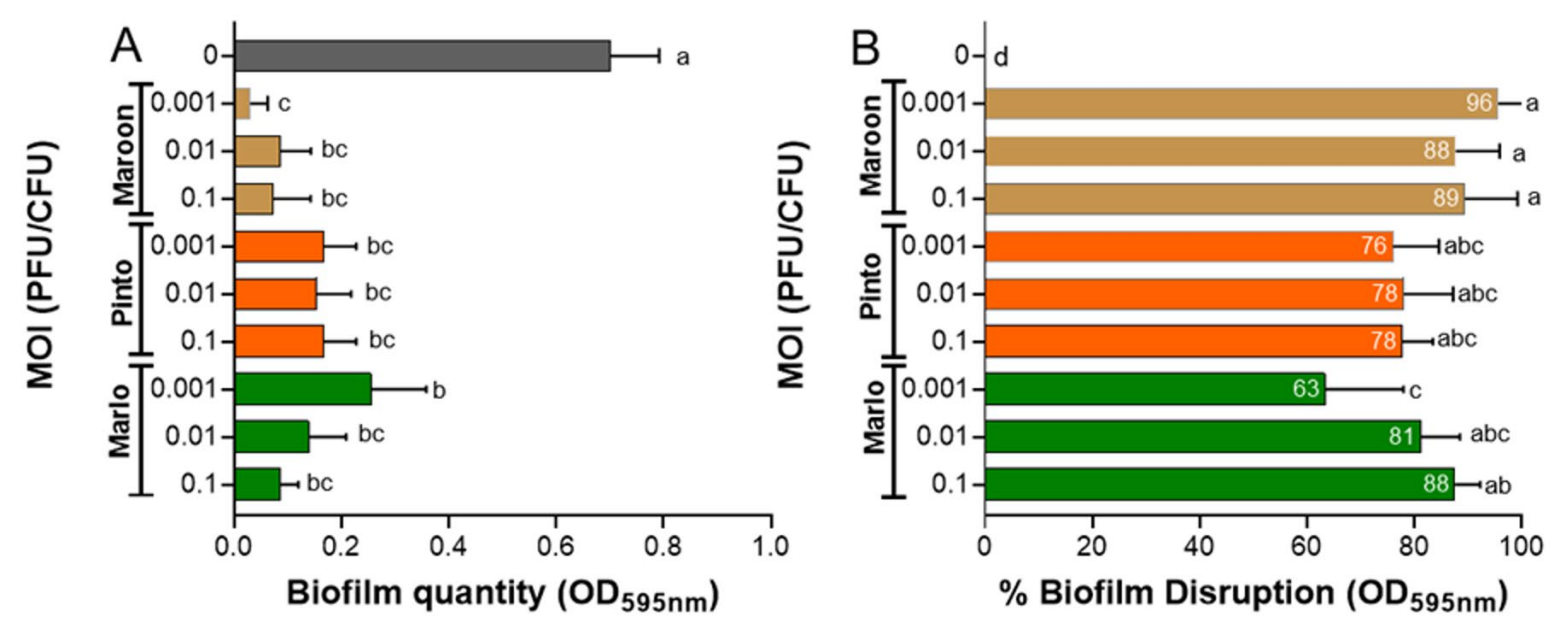
Phage-mediated disruption of planktonic and biofilm cells of almond-infecting *Pseudomonas syringae* pv. *syringae* 2507. (**A**) Biofilm biomass following treatment with phages vB_PsyP_Mobley, vB_PsyP_Plaza, and vB_PsyP_Mission at the indicated MOIs, quantified by 0.1% crystal violet staining and measured at 595 nm. (**B**) Percentage reduction in biofilm biomass relative to untreated controls. Data are presented as mean ± SD from three technical replicates and at least two independent experiments. Statistical significance was determined using one-way ANOVA followed by Tukey’s multiple comparisons test. Different lowercase letters indicate statistically significant differences among treatments (*p* < 0.05).

The bactericidal activity of phages vB_PsyP_Mobley, vB_PsyP_Plaza, and vB_PsyP_Mission against planktonic *Pseudomonas* cells was assessed by monitoring optical density (OD600) over time. All three phages exhibited MOI-dependent reductions in bacterial density during the early phase of infection. vB_PsyP_Mobley reduced OD600 within 4–8 h across MOIs from 0.00001 to 1, with more rapid decline at higher MOIs; at an MOI of 10, OD600 dropped to near baseline within 2 h. However, bacterial regrowth consistent with the emergence of resistant cells was observed by 16 h (Fig. 3A, D). vB_PsyP_Plaza caused a rapid early decrease in OD600 (within ~ 2 h) across MOIs ranging from 10⁻⁹ to 10⁻², followed by regrowth at later time points (Fig. 3B, E). vB_PsyP_Mission showed a similar pattern, with a pronounced early reduction in OD600 within ~ 2 h across the same MOI range and subsequent regrowth at later time points (Fig. 3C, F). Both vB_PsyP_Plaza and vB_PsyP_Mission retained measurable activity at very low MOIs, whereas vB_PsyP_Mobley showed its strongest early activity at MOIs ≤ 1. All three phages also disrupted pre-formed biofilms. When applied at MOIs from 0.1 to 0.001, vB_PsyP_Mobley, vB_PsyP_Plaza, and vB_PsyP_Mission reduced biofilm biomass by 63–88%, 76–78%, and 88–96%, respectively, as measured by crystal violet staining (Fig. 4A, B). vB_PsyP_Mission consistently produced the greatest reduction, consistent with its halo-forming plaque morphology, which suggests EPS-degrading activity. Together, these results indicate that vB_PsyP_Mobley, vB_PsyP_Plaza, and vB_PsyP_Mission produce strong early bactericidal effects against planktonic cells and significantly reduce established biofilms, with differences in activity profiles across MOIs and in biofilm disruption.

### Stability and adsorption kinetics of phage

**Fig. 5:**
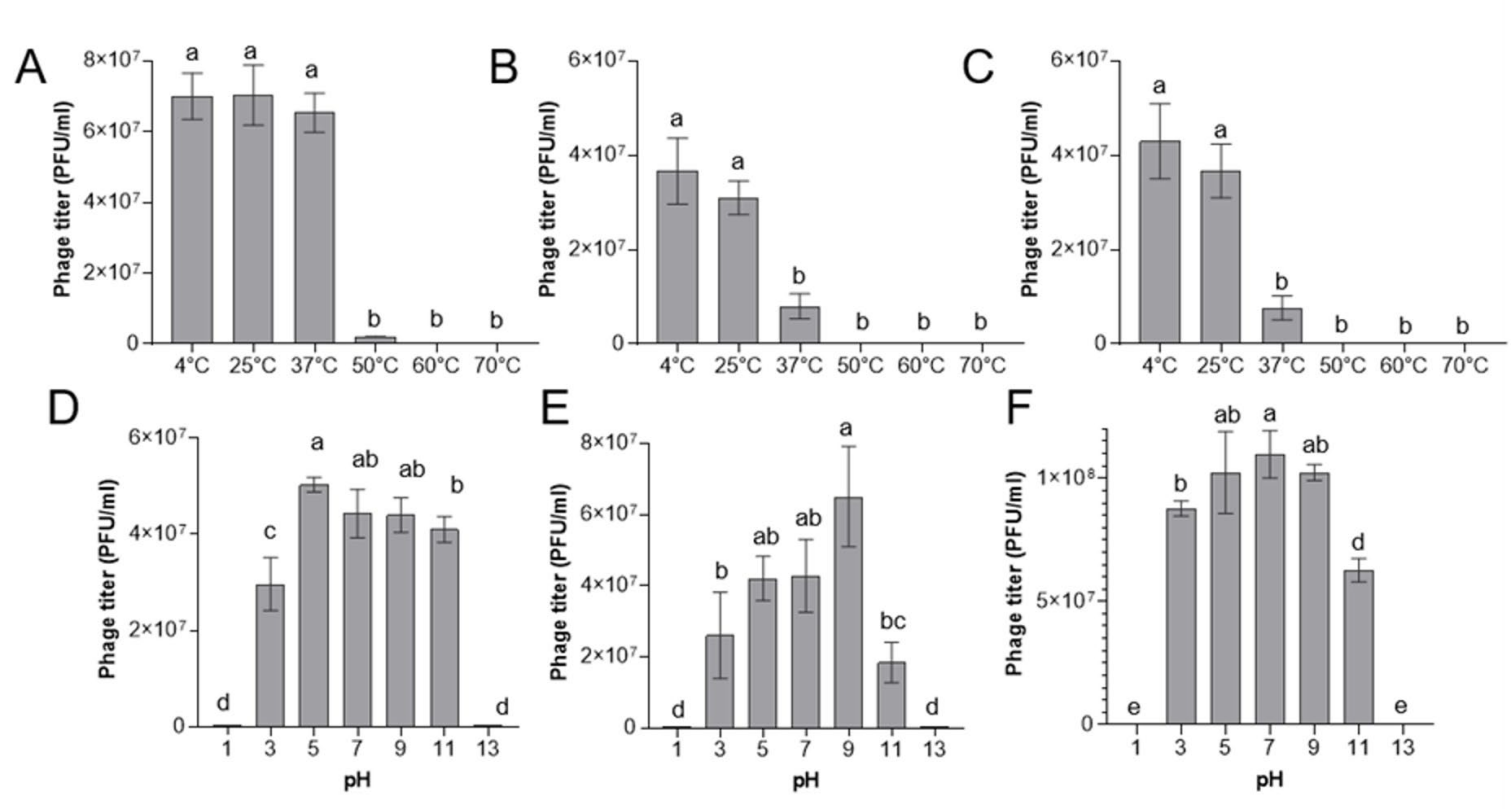
Effect of temperature and pH on the viability of almond-infecting *Pseudomonas syringae* pv. *syringae* 2507 phages. (**A–C**) Viability of phages vB_PsyP_Mobley (**A**), vB_PsyP_Plaza (**B**), and vB_PsyP_Mission (**C**) following 24 h incubation at the indicated temperatures. (**D–F**) Viability of vB_PsyP_Mobley (**D**), vB_PsyP_Plaza (**E**), and vB_PsyP_Mission (**F**) following 1 h incubation at the indicated pH values. Phage viability is expressed as PFU/mL. Data represent mean ± SD from at least two independent experiments. Statistical comparisons were performed using one-way ANOVA with Tukey’s multiple comparisons test. Bars sharing the same lowercase letter are not significantly different (*p* < 0.05).

**Fig. 6:**
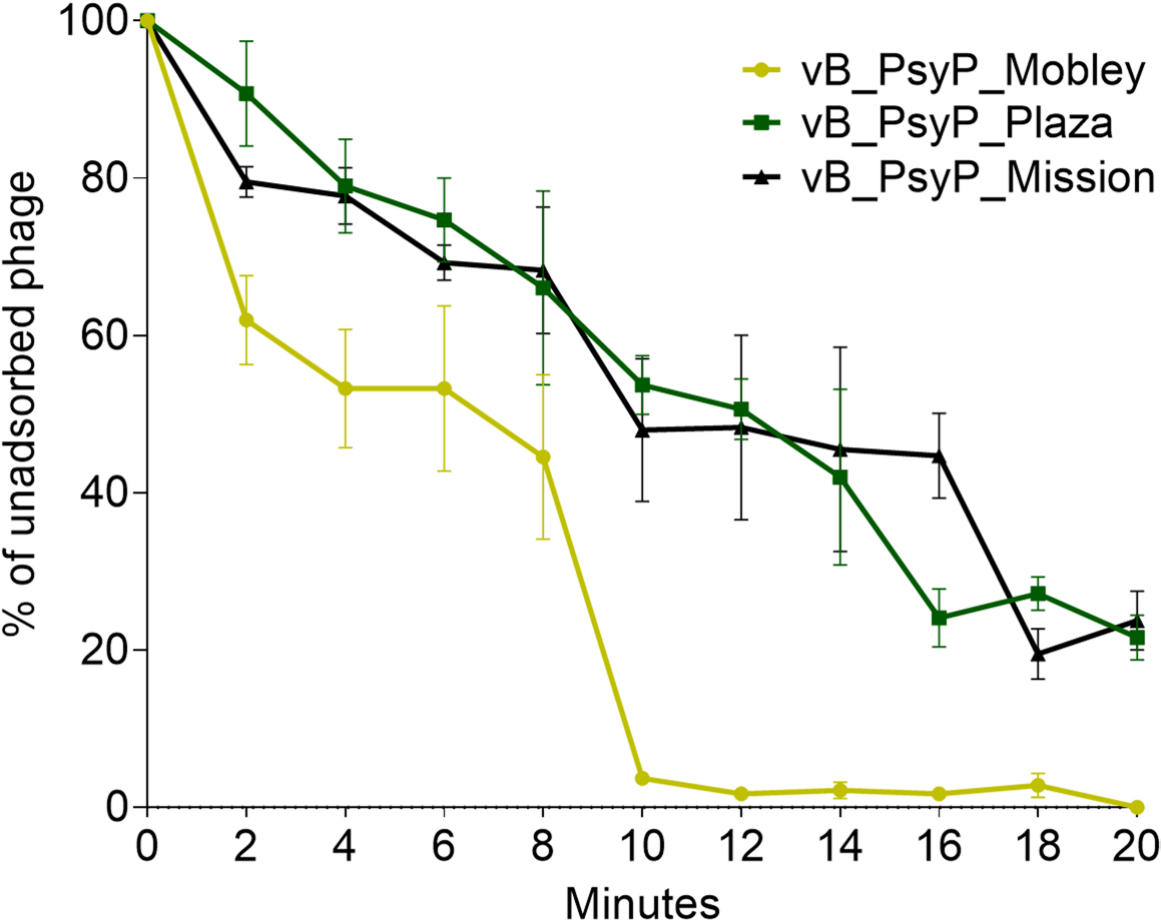
Adsorption kinetics of almond-infecting *Pseudomonas syringae* pv. *syringae* 2507 phages. Adsorption curves of phages vB_PsyP_Mobley, vB_PsyP_Plaza, and vB_PsyP_Mission were determined by mixing each phage with host cells at multiplicities of infection (MOI) of 0.001 for vB_PsyP_Mobley and vB_PsyP_Plaza, and 0.0001 for vB_PsyP_Mission. At defined time intervals, aliquots were removed, and the number of unadsorbed phages was quantified by plaque assay.

Temperature and pH stability are important considerations for field application of phages, as environmental conditions can influence infectivity. Thermal stability assays revealed that vB_PsyP_Mobley, vB_PsyP_Plaza, and vB_PsyP_Mission remained viable after 24 h of incubation at between 4 °C and 25 °C (Fig. 5A–C). This stability is consistent with the cold spring conditions under which almond bacterial blast commonly develops^3,7,8,23^. Phage vB_PsyP_Mobley retained infectivity at 37 °C, whereas phages vB_PsyP_Plaza and vB_PsyP_Mission showed marked reductions in activity at this temperature. At 50 °C and above, infectivity of all three phages was minimal or undetectable. Across pH treatments, all phages maintained infectivity over a broad range (pH 3–11), although with phage-specific profiles (Fig. 5D–F). Extreme acidic (pH 1) or alkaline (pH 13) conditions eliminated detectable infectivity. Among the three phages, vB_PsyP_Mobley exhibited comparatively greater thermal tolerance, while vB_PsyP_Mission showed broader stability across the tested pH range, suggesting differences in capsid or virion structural resilience. Adsorption assays further distinguished the phages’ infection dynamics. vB_PsyP_Mobley adsorbed to *Pseudomonas* cells more rapidly than vB_PsyP_Plaza and vB_PsyP_Mission, which displayed similar but slower adsorption kinetics (Fig. 6). Together, these results indicate that the three phages differ in their stability and early infection traits, with vB_PsyP_Mobley combining faster host adsorption and greater thermal tolerance, and vB_PsyP_Plaza and vB_PsyP_Mission exhibiting moderate stability and slower adsorption.

### Genomic features and assembly quality of vB_PsyP phages

The complete genomes of phages vB_PsyP_Mobley, vB_PsyP_Plaza, and vB_PsyP_Mission were sequenced using the Illumina MiSeq platform and assembled into single, high-quality contigs. Genome sizes were 39,888 bp for vB_PsyP_Mobley, 41,879 bp for vB_PsyP_Plaza, and 40,099 bp for vB_PsyP_Mission, with GC contents of 63%, 57%, and 56%, respectively (Table 5). Assembly quality assessment using CheckV and MiUVIG criteria confirmed that all three genomes were complete, with 100% estimated completeness and average amino acid identity (AAI)–based confidence values exceeding 90%. All three genomes contained direct terminal repeats (DTRs) of identical length (87 bp; repeat count = 2), indicating a conserved DTR-mediated DNA packaging strategy. Functional annotation identified 58 open reading frames (ORFs) in vB_PsyP_Mobley, 54 in vB_PsyP_Plaza, and 51 in vB_PsyP_Mission (Table 6). Canonical modules for DNA replication, morphogenesis, and host lysis were present in all genomes, whereas integrases, recombination modules, tRNAs, CRISPR arrays, virulence-associated genes, and antimicrobial resistance determinants were absent, indicating that these phages do not establish lysogeny. vB_PsyP_Mobley encoded the highest proportion of hypothetical or uncharacterized ORFs (34), followed by vB_PsyP_Plaza (23) and vB_PsyP_Mission (20), suggesting differing degrees of functional novelty among the phages.

**Table 5:** Genome assembly metrics of phages vB_PsyP_Mission, vB_PsyP_Plaza, and vB_PsyP_Mobley.

Assembly metrics	vB_PsyP_Mobley	vB_PsyP_Plaza	vB_PsyP_Mission
Contig length (bp)	41,879	40,099	39,888
Accession numbers	PX694326	PX694327	PX694328
Coverage	301x	277x	239x
% GC	63	57	56
CheckV quality	Complete	Complete	Complete
MIUVIG quality	High-quality	High-quality	High-quality
Completeness (%)	100	100	100
Packaging mechanism	Direct terminal repeats	Direct terminal repeats	Direct terminal repeats
Confidence level	high	high	high
Confidence reason	AAI-based completeness > 90%	AAI-based completeness > 90%	AAI-based completeness > 90%
Repeat length	87	87	87
Repeat count	2	2	2
Repeat sequence	TGACCACTCTCGCACTGGCTGGCGCTATGTGGCACTGCTACAAGTCCAACAAGCCACTCCGTGAGAAAGCCCGTGAAGAGGCCGAAG	GCTCAGGCAGACGCAGCATCGGCTAACGCCCTGCAAATCGCCAACGCCGCCAAGGCCACGGCTGAGGGCATCGACGCGAAAGCCACC	CCTCATTGAGTAGCCCATAATGATCCCCATGAGATGCCCTCGAAGGAACCGAGGCGATCATGCTCGAAGGGTCACCTGGGAGAGGCC

**Table 6:** Comparative genomic features and functional annotation of phages vB_PsyP_Mobley, vB_PsyP_Plaza, and vB_PsyP_Mission.

Description	vB_PsyP_Mobley	vB_PsyP_Plaza	vB_PsyP_Mission
CDS	58	54	51
Connector	1	1	1
DNA, RNA and nucleotide metabolism	8	10	10
Head and packaging	7	8	9
Integration and excision	0	0	0
Lysis	2	3	3
Moron, auxiliary metabolic gene and host takeover	2	3	3
Other	0	1	1
Tail	4	4	4
Transcription regulation	0	0	0
Unknown function	34	23	20
tRNAs	0	0	0
CRISPRs	0	0	0
Virulence factors	0	0	0
Anti-microbial resistance genes	0	0	0
Coding density	97.08	95.29	95.37

### Genome-based classification and taxonomic placement

Genome-based classification using ICTV-recognized taxonomy frameworks placed all three phages within the realm *Duplodnaviria*, kingdom *Heunggongvirae*, phylum *Uroviricota*, class *Caudoviricetes*, and order *Autographivirales*. All were assigned to the family *Autotranscriptaviridae* (Table 7). Subfamily- and genus-level resolution differed among the phages. vB_PsyP_Plaza and vB_PsyP_Mission were placed within the subfamily *Studiervirinae*, clustering at the genus level within *Ghunavirus* and *Pifdecavirus*, respectively. In contrast, vB_PsyP_Mobley could not be confidently assigned to a defined subfamily or genus and is provisionally designated as unclassified within *Autotranscriptaviridae*. Whole-genome nucleotide comparisons supported these placements. Pairwise intergenomic identities among vB_PsyP_Mobley, vB_PsyP_Plaza, and vB_PsyP_Mission ranged from 40.93% to 45.56% (Table 8), indicating that the three phages represent distinct evolutionary lineages relative to one another. Comparison with previously described phages (Table 9) showed that vB_PsyP_Mission shares 96.08% identity (98% coverage) with *Pseudomonas* phage vB_PpuP-Keila, and vB_PsyP_Plaza shares 97.91% identity (97% coverage) with *Pseudomonas* phage Pst_gh1. According to ICTV species demarcation thresholds (≥ 95% genome-wide nucleotide identity), these values indicate that vB_PsyP_Mission and vB_PsyP_Plaza represent novel isolates within previously established species-level groups rather than new species. In contrast, vB_PsyP_Mobley exhibits lower similarity to its closest reference, *Pseudomonas* phage KBC54 (83.87% identity; 80% coverage), consistent with greater genomic divergence.

**Table 7:** ICTV-based taxonomic placement of vB_PsyP_Mobley, vB_PsyP_Plaza, and vB_PsyP_Mission inferred from genomic and phylogenetic analyses.

Genome	vB_PsyP_Mobley	vB_PsyP_Plaza	vB_PsyP_Mission
Realm	Duplodnaviria	Duplodnaviria	Duplodnaviria
Kingdom	Heunggongvirae	Heunggongvirae	Heunggongvirae
Phylum	Uroviricota	Uroviricota	Uroviricota
Class	Caudoviricetes	Caudoviricetes	Caudoviricetes
Order	Autographivirales	Autographivirales	Autographivirales
Family	Autotranscriptaviridae	Autotranscriptaviridae	Autotranscriptaviridae
Subfamily	Unknown	Studiervirinae	Studiervirinae
Genus	New genus	Ghunavirus	Pifdecavirus
Species	New species	Ghunavirus (new name)	Pifdecavirus (new name)

**Table 8:** Percentage intergenomic identity matrix for Phages vB_PsyP_Mobley, vB_PsyP_Plaza, and vB_PsyP_Mission.

Phage Pair	Percent Identity (%)
vB_PsyP_Mobley × vB_PsyP_Mobley	100.00
vB_PsyP_Mobley × vB_PsyP_Mission	45.56
vB_PsyP_Mobley × vB_PsyP_Plaza	41.40
vB_PsyP_Mission × vB_PsyP_Mission	100.00
vB_PsyP_Mission × vB_PsyP_Plaza	40.93
vB_PsyP_Plaza × vB_PsyP_Plaza	100.00

**Table 9:** Pairwise comparison table for Phages vB_PsyP_Mobley, vB_PsyP_Plaza, and vB_PsyP_Mission, and their closely-related phages.

Features	vB_PsyP_Mobley	vB_PsyP_Plaza	vB_PsyP_Mission
Accession	OL854071.1	LC730319	PP496426.1
Description	*Pseudomonas* Φ KBC54	*Pseudomonas* Φ gh-1	*Pseudomonas* Φ vB_PpuP-Keila
Genome length (bp)	42,609	40,485	40,268
% Querry coverage	80	97	98
% Identity	83.87	97.91	96.08
Isolation source	Soil	Lettuce infected leaves	Clear river water
Isolation host	*P. syringae* pv. *actinidiae*	*P. syringae* pv. *tomato*	*P. putida*
Isolation location	China	France	Estonia, Europe

### Phylogenomic relationships and genome organization

**Fig. 7:**
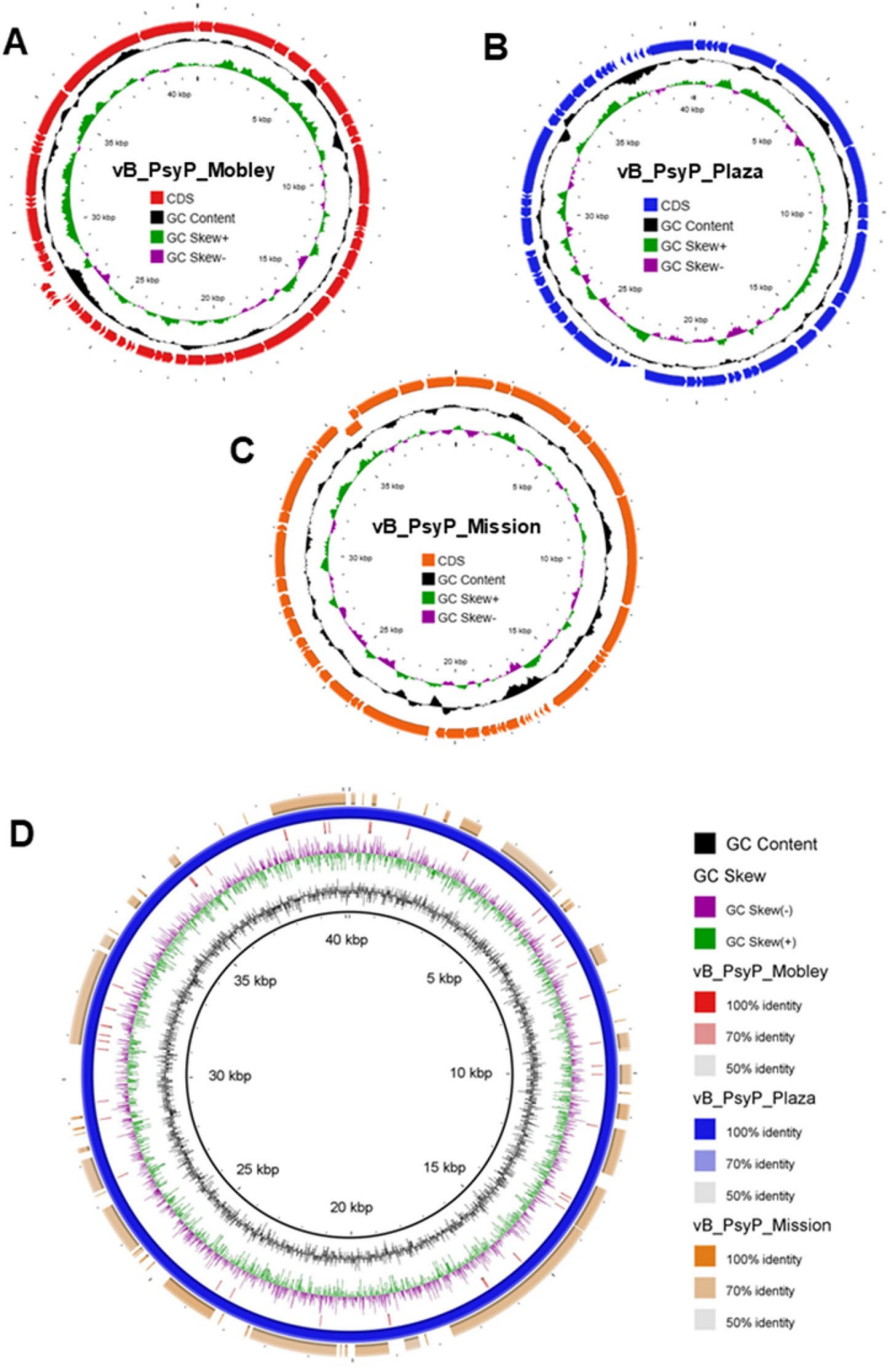
Genomic architecture and whole-genome similarity of three almond-infecting vB_PsyP phages. (**A–C**) Circular genome maps of phages vB_PsyP_Mobley (**A**), vB_PsyP_Plaza (**B**), and vB_PsyP_Mission (**C**). Predicted coding sequences (CDS) are shown on the outer ring (color-coded by phage), with inner rings indicating GC content (black) and GC skew (green = positive; purple = negative). (**D**) BRIG-based whole-genome comparison of vB_PsyP_Mobley, vB_PsyP_Plaza, and vB_PsyP_Mission, with shaded rings indicating nucleotide identity thresholds (50%, 70%, and 100%) relative to the reference genome.

**Fig. 8:**
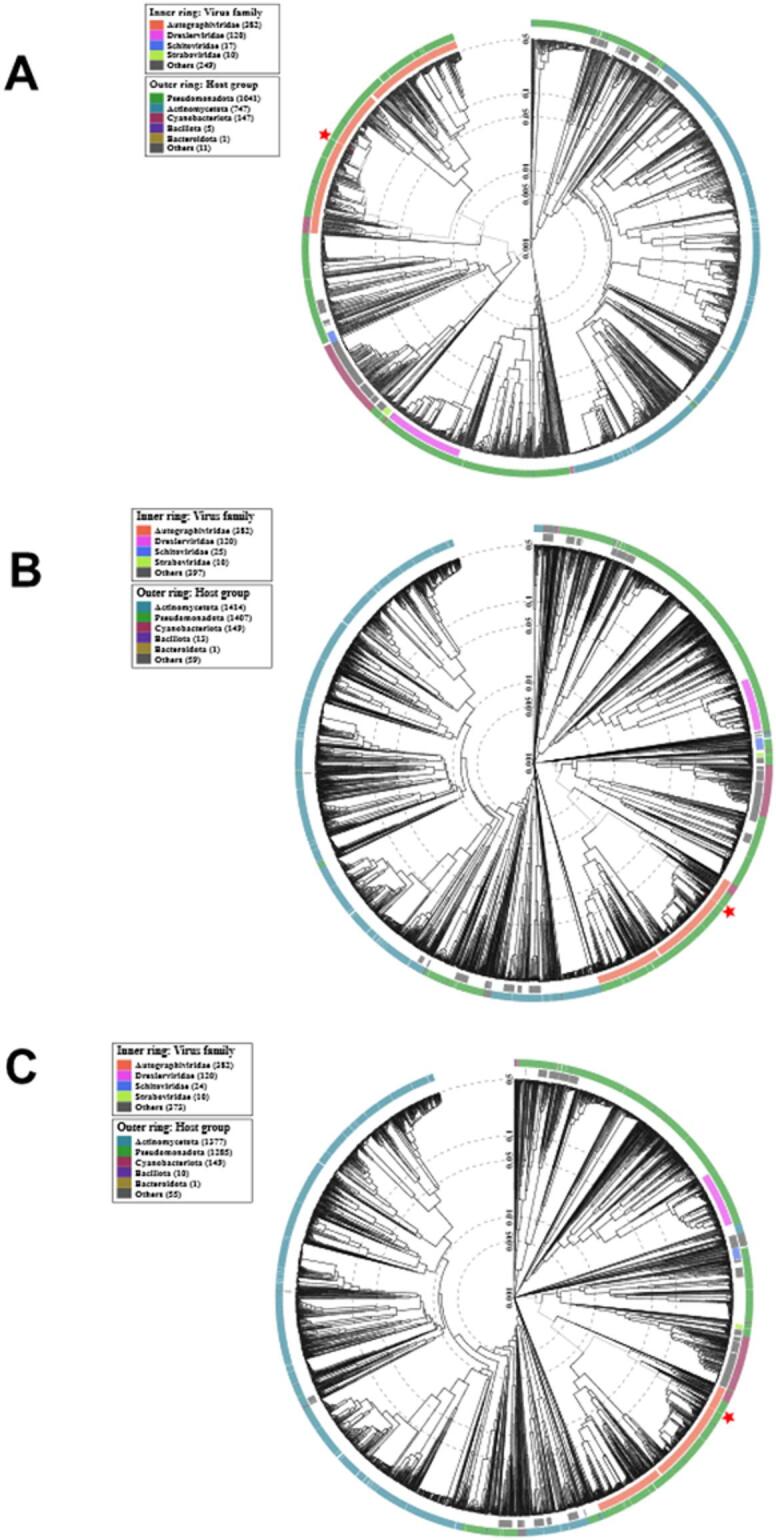
Phylogenetic analysis of almond-infecting *Pseudomonas syringae* pv. *syringae* 2507 phages. Phylogenetic tree generated using ViPTree based on whole-genome proteomic alignments of phages vB_PsyP_Mobley (**A**), vB_PsyP_Plaza (**B**), and vB_PsyP_Mission (**C**) with related phages from established reference genomes.

**Fig. 9:**
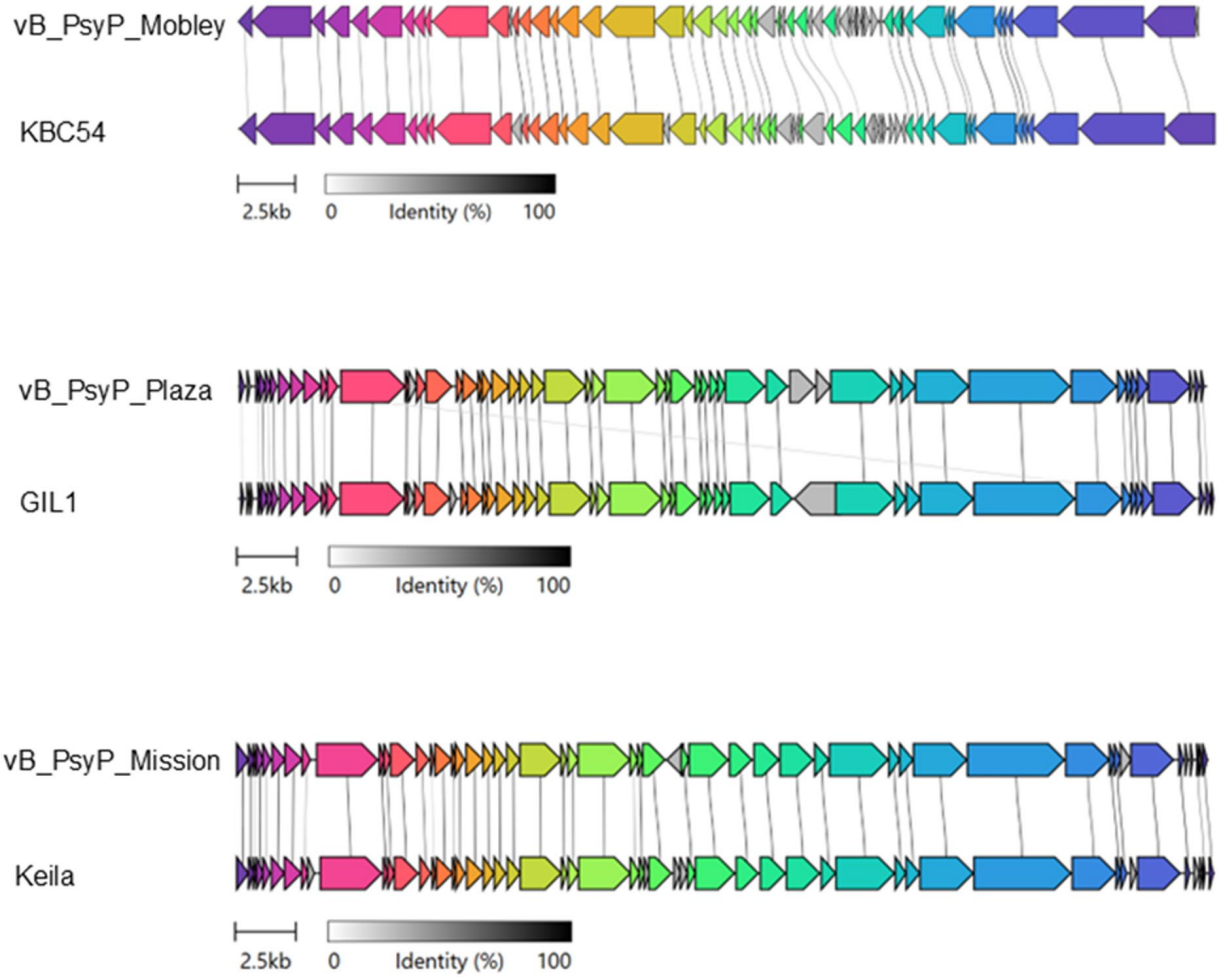
Comparative genomic architecture of phages vB_PsyP_Mission, vB_PsyP_Plaza, and vB_PsyP_Mobley and closely-related phages. Gene synteny and amino acid identity are shown for each phage and its reference counterpart: (**A**) vB_PsyP_Mobley vs. KBC54, (**B**) vB_PsyP_Plaza vs. GIL1, and (**C**) vB_PsyP_Mission vs. Keila. Colored arrows represent predicted ORFs, and grey lines indicate homologous regions. The identity scale ranges from 0–100%.

**Fig. 10:**
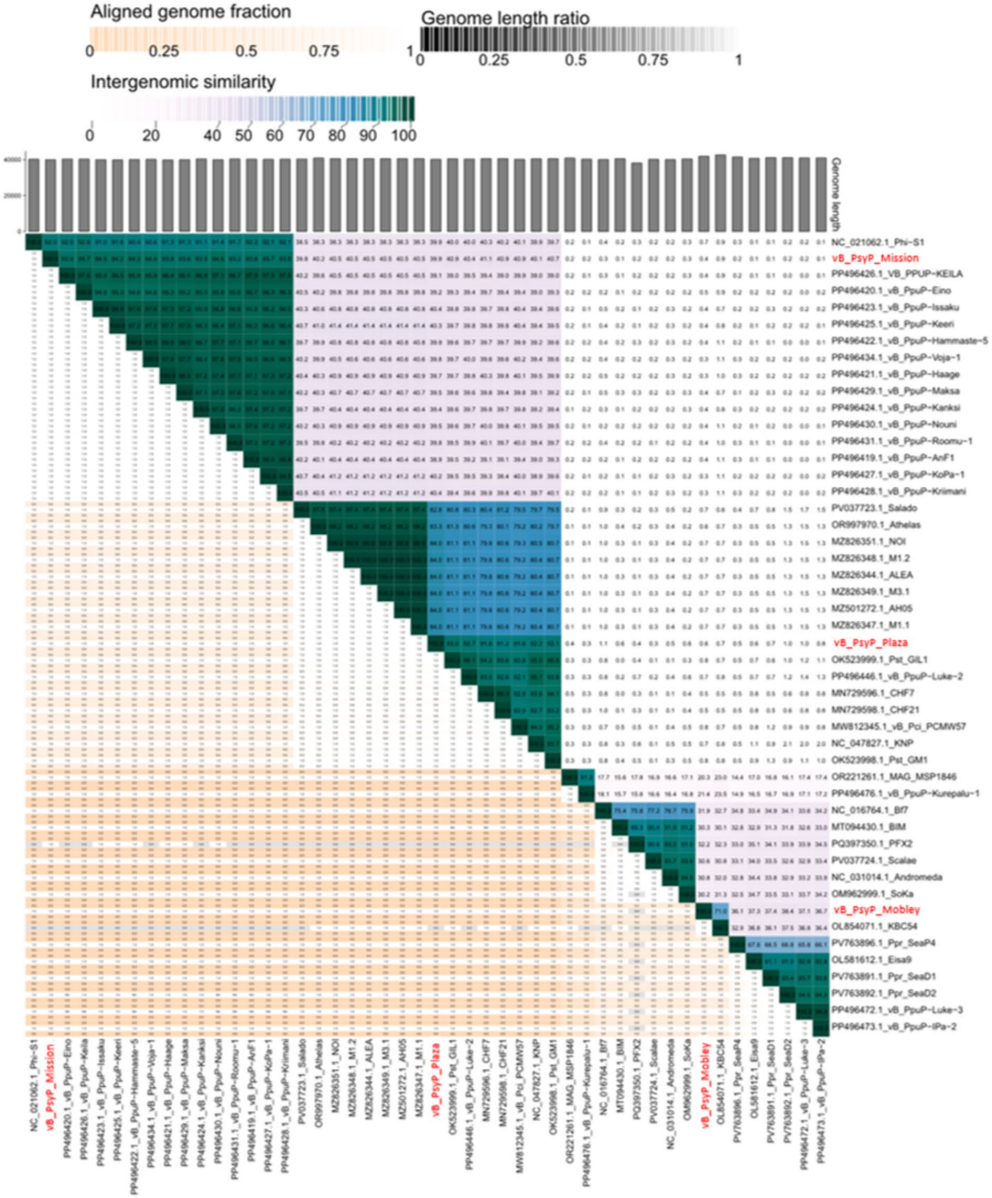
VIRIDIC pairwise intergenomic similarities and distances among phages vB_PsyP_Mobley, vB_PsyP_Plaza, and vB_PsyP_Mission. Color density represents aligned genome fraction, genome length ratio, and intergenomic similarity values, where darker shading corresponds to lower similarity or limited alignment. Gray bars indicate relative genome lengths. The names of the three focal phages are bolded and shown in red for emphasis.

Phylogenetic analyses based on amino acid sequences of two conserved structural markers, including the major capsid protein (MCP, Fig. S1) and terminase large subunit (TerL, Fig. S2), further resolved these relationships. Both trees showed congruent topologies. vB_PsyP_Plaza clustered within Group I alongside Pst_gh1-related phages, vB_PsyP_Mobley clustered within Group II with KBC54-related phages, and vB_PsyP_Mission clustered within Group III with vB_PpuP-Keila and related representatives. A separate Group IV comprised more distantly related *Pseudomonas* and *EctoPseudomonas* phages. In both trees, the *Pectobacterium* phages used as outgroups were clearly separated from the *Pseudomonas* clades. The consistent grouping across MCP and TerL phylogenies supports the lineage assignments inferred from whole-genome analyses. Phylogenomic clustering using ViPTree (Figs. 7, 8 A-C) produced similar relationships, with vB_PsyP_Plaza and vB_PsyP_Mission clustering closely with their respective reference phages, whereas vB_PsyP_Mobley formed a more distant branch. Whole-genome synteny analysis further reflected these patterns. vB_PsyP_Plaza and Pst_gh1, as well as vB_PsyP_Mission and vB_PpuP-Keila, exhibited extensive collinearity across structural, replication, and lysis modules. In contrast, vB_PsyP_Mobley shared only partial synteny with KBC54, with several divergent regions enriched in unique ORFs and hypothetical genes (Fig. 9). VIRIDIC intergenomic similarity analysis (Fig. 10) also showed that vB_PsyP_Mission and vB_PsyP_Plaza cluster with multiple closely related *Pseudomonas* phages, whereas vB_PsyP_Mobley clustered with only KBC54, further highlighting its relative genomic distinctness. Circular genome maps (Fig. 7A-C) showed densely packed coding regions and characteristic GC content and GC skew patterns corresponding to structural and replication modules, consistent with modular genome organization typical of tailed dsDNA phages.

### Comparative gene content and predicted depolymerase repertoire

Comparative gene content analysis demonstrated that vB_PsyP_Plaza and vB_PsyP_Mission share a substantial proportion of orthologous genes, whereas vB_PsyP_Mobley shares fewer conserved genes with the other two phages (Fig. 7D; Fig. S3; Tables S2-S4). Although all three genomes retain conserved structural and replication backbones, each contains regions enriched in unique ORFs, with vB_PsyP_Mobley exhibiting the greatest divergence. Reciprocal BLASTP-based ortholog comparisons (Tables S5-S7) identified conserved structural and replication modules across the three genomes but revealed divergence in genes associated with host interaction and infection processes. Sequence variation was observed in tail fiber proteins, tail-associated proteins, head-tail adaptor components, and infection-related enzymes, including DNA polymerase, RNA polymerase, DNA ligase, and DNA primase/helicase. Differences were also detected in holin, Rz-like spanin, internal virion proteins, and a predicted host HNS inhibition protein, indicating variability in infection-related gene repertoires among the lineages. Depolymerase prediction analysis identified putative virion-associated enzymatic candidates in all three genomes (Table 10). Each phage encoded a predicted tail-associated depolymerase (YCJ12281.1_41 in vB_PsyP_Mobley; YCJ12353.1_55 in vB_PsyP_Plaza; YCJ12402.1_49 in vB_PsyP_Mission). In addition, vB_PsyP_Mobley encoded a second predicted depolymerase annotated as an internal virion protein, whereas vB_PsyP_Plaza and vB_PsyP_Mission each encoded an additional predicted depolymerase annotated as a tail fiber protein. These predicted candidates varied in sequence length and physicochemical properties among the genomes. All depolymerase assignments remain bioinformatic predictions pending biochemical validation.

**Table 10:** Comparative depolymerase predictions of phages vB_PsyP_Mobley, vB_PsyP_Plaza, and vB_PsyP_Mission.

Phage	Locus tag	Annotation	Probability	Size (bp)	Aromaticity	Instability index	Isoelectric point	GRAVY
Mobley	YCJ12281.1_41	Tail protein	0.95	2573	0.10	31.25	5.63	−0.19
	YCJ12278.1_38	Internal virion protein	0.86	3704	0.06	32.15	6.05	−0.40
Plaza	YCJ12353.1_55	tail protein	0.91	1964	0.12	34.59	4.94	−0.24
	YCJ12348.1_50	tail fiber protein	0.91	1883	0.07	34.82	6.56	−0.31
Mission	YCJ12402.1_49	tail protein	0.90	2420	0.11	34.13	5.40	−0.27
	YCJ12355.1_2	tail fiber protein	0.82	1787	0.07	24.63	6.33	−0.42

## Discussion

This study provides an integrated phenotypic, ecological, genomic, and phylogenomic characterization of three strictly lytic phages infecting *P. syringae* associated with almond bacterial blast. While *in planta* efficacy was not evaluated, the data define key attributes relevant to agricultural deployment, including phylogenetically structured host range, quantitative infectivity (EOP), infection dynamics, environmental stability, genome organization, and taxonomic placement. By comparing three evolutionarily distinct phages with overlapping but non-identical biological traits, this work illustrates how lineage-level diversity may be leveraged to address pathogen heterogeneity in perennial cropping systems.

A central finding is that all three phages exhibit their strongest infectivity against almond-associated *P. syringae* pv. *syringae* isolates belonging to phylogroup 2 (PG2), whereas activity against PG7 (*P. viridiflava*) is reduced and often partial. Importantly, host range conclusions are supported by quantitative EOP measurements in addition to plaque morphology. Framing host range in a phylogenetic context is particularly important in *Pseudomonas*, where phylogroups frequently reflect underlying differences in surface architecture, including lipopolysaccharide O-antigen composition, outer membrane proteins, and extracellular polysaccharides^24,25^. The PG-associated infectivity patterns observed here are therefore consistent with receptor conservation within PG2 and receptor divergence in PG7 affecting adsorption efficiency, post-entry compatibility, or replication dynamics. This aligns with broader evidence that phage host range often tracks receptor phylogeny more closely than formal species or pathovar boundaries^26^. From a disease management standpoint, this suggests that phage selection can be optimized for dominant outbreak lineages—such as PG2 almond-associated strains—while recognizing that coverage of more distant clades may require complementary isolates or engineered receptor-binding variants^27^.

Phylogenomic and whole-genome analyses further contextualize these ecological patterns. Despite low intergenomic nucleotide identity among vB_PsyP_Mobley, vB_PsyP_Plaza, and vB_PsyP_Mission, all three infect PG2 populations, suggesting that distinct evolutionary lineages can independently access the same ecological niche through different structural or receptor-binding configurations^28,29^. Previous work has shown that infection outcomes are shaped by lineage-specific combinations of receptor-binding proteins, tail structures, and accessory genes that influence adsorption, intracellular compatibility, and lysis dynamic^16,30^. Whole-genome identity thresholds indicate that Plaza and Mission represent new isolates within previously defined species-level groups, whereas Mobley is more divergent and potentially represents a distinct lineage. Based on its comparatively low nucleotide identity to currently described relatives, limited clustering with reference phages, and concordant placement as a distinct lineage across MCP, TerL, synteny, and VIRIDIC analyses, vB_PsyP_Mobley may represent a member of a previously unrecognized genus. However, formal genus designation will require broader comparative analysis and validation under ICTV taxonomic criteria. Concordant placement across MCP and TerL phylogenies, genome synteny, and VIRIDIC clustering strengthens these assignments and supports their evolutionary distinctness. From an applied perspective, genomic distinctness combined with overlapping host coverage may be advantageous for cocktail design, as resistance often arises through receptor modification or intracellular defenses^31–34^. However, genomic divergence alone does not confirm independent receptor usage, as unrelated phages can converge on the same surface determinant^35,36^. Accordingly, while the present data support functional complementarity, definitive receptor mapping and cross-resistance testing are required to confirm distinct entry pathways.

The concordance between halo formation, biofilm disruption, and predicted depolymerase-like genes provides an additional functional dimension often lacking in phage biocontrol studies^37,38^. In this study, vB_PsyP_Mission consistently produced plaques surrounded by halos, whereas vB_PsyP_Mobley and vB_PsyP_Plaza did not. In *Pseudomonas*, extracellular matrices can include alginate-like polymers, levan, cellulose-associated components, and other strain- and environment-dependent EPS structures^39–42^. Virion-associated depolymerases facilitate penetration of these barriers and can improve access to surface receptors^43,44^. Interpreted conservatively, the concordance between halo formation in vB_PsyP_Mission and the presence of predicted depolymerase-like genes is consistent with a model in which matrix modification enhances access to bacterial cells embedded within structured environments, whereas vB_PsyP_Mobley and vB_PsyP_Plaza may rely primarily on lytic replication against more accessible targets^45^. From a biocontrol standpoint, this suggests potential ecological differentiation rather than superiority. Halo-forming phages may contribute to disruption of surface-associated microcolonies, whereas non-halo-forming phages may more efficiently target exposed populations^46,47^. In perennial systems such as almond orchards, where epiphytic persistence and microenvironmental heterogeneity are common, such complementary roles could be advantageous. Nonetheless, halo formation alone does not predict field efficacy, and biochemical validation of putative depolymerases remains necessary to confirm substrate specificity and functional relevance.

The shared presence of direct terminal repeats (DTRs) across all three phages, despite broader genomic divergence, highlights conservation of packaging architecture independent of lineage identity. DTR-mediated packaging imposes constraints on terminase function and capsid–genome interactions^48,49^. In this sense, DTR conservation represents an architecture-level similarity, indicating that distinct phage lineages infecting similar hosts can converge on comparable solutions for genome replication and packaging while differing substantially in host-recognition modules and accessory gene content^50^. It is noteworthy that, although this feature does not directly inform lytic efficacy, host range, or performance in combined applications, nor does it inherently imply interaction or interference among phages^48^, it nevertheless provides insight into conserved replication and packaging architectures shared across otherwise divergent phage lineages.

Differences in thermal tolerance among the phages have practical relevance for field deployment. The retention of infectivity by vB_PsyP_Mobley at 37 °C, compared with reduced stability of Plaza and Mission, suggests that environmental conditions and application timing may influence relative performance^51,52^. Almond bacterial blast typically develops under cool, wet conditions, but short-term temperature elevations above 30 °C are common in the Central Valley^7,8^. Under such conditions, Mobley may maintain activity more reliably, whereas Plaza and Mission are likely to perform best when applications are aligned with cooler windows, such as early morning or evening periods. In addition to temperature, pH is another field-relevant factor that may influence phage persistence. Although orchard conditions are not defined by extreme pH, phage exposure to variable pH can occur on leaf surfaces, particularly via irrigation water quality, tank mixes, and formulation additives. Consistent with our stability assays across a broad pH range, these results suggest that pH dynamics, together with temperature, should be considered when optimizing formulation and application conditions for orchard deployment.

In conclusion, this study provides an integrated characterization of three strictly lytic phages (vB_PsyP_Mobley, vB_PsyP_Plaza, and vB_PsyP_Mission) infecting almond-associated *P. syringae* pv. *syringae* and establishes a foundation for developing phage-based management strategies. However, receptor identification and mechanistic analyses explaining cross-phylogroup infectivity remain priorities for future work. In addition, key assays were performed on a limited set of strains, and broader isolate-level robustness remains to be evaluated. Environmental stability was assessed under controlled conditions, and additional orchard-relevant stressors, including UV exposure and fluctuating humidity, were not tested. Finally, all experiments were conducted in vitro, and phage activity under epiphytic and *in planta* conditions remains to be determined. Collectively, these results define a genetically diverse set of strictly lytic phages with distinct infection traits and provide a strong platform for subsequent quantitative, mechanistic, and in planta/field-based evaluations. Notably, the degree of genomic and phylogenetic divergence observed for vB_PsyP_Mobley suggests that it may warrant future consideration as a representative of a previously unrecognized genus, pending formal taxonomic validation.

## Materials and methods

### Phage isolation and purification

Soil samples collected from the vicinity of Sproul Hall at UC Riverside and sewage samples obtained from the Riverside wastewater treatment facility were collected and processed for bacteriophage isolation. Samples were mixed with an equal volume of 2× LB medium and incubated at 28 °C with shaking for 4 h to facilitate the release of phages into solution. Following incubation, samples were clarified by centrifugation and passed through 0.22 μm syringe filters to remove bacterial debris. For phage enrichment, 10 mL of the filtered sample was combined with 20 mL of actively growing cultures of the target *Pseudomonas* strains and incubated overnight at 28 °C with shaking at 200 rpm. Enrichment cultures were then centrifuged at 3,500 rpm for 10 min, and the supernatants were filtered through 0.22 μm syringe filters. Phage activity was initially screened using spot assays. Briefly, 100 µL of each bacterial culture was mixed with 5 mL of molten LB soft agar (0.5% agar) and overlaid onto LB agar plates to form bacterial lawns. Aliquots (10 µL) of enriched phage suspensions were spotted onto the lawns, and plates were incubated overnight at 28 °C. Zones of lysis were identified and individual plaques were collected using sterile pipette tips and resuspended in SM buffer (50 mM Tris-HCl, 100 mM NaCl, 8 mM MgSO₄, 0.01% gelatin). To ensure clonal purity, phages were purified through three successive rounds of plaque isolation. High-titer phage stocks were prepared and stored at 4 °C for short-term use or at − 80 °C in SM buffer supplemented with 20% glycerol for long-term preservation.

### Host range determination and efficiency of plating

The host range of phages vB_PsyP_Mobley, vB_PsyP_Plaza, and vB_PsyP_Mission was evaluated using a panel of 36 *Pseudomonas* strains representing multiple species and pathovars associated with diverse plant hosts. Almond-associated *P. syringae* pv. *syringae* isolates were obtained from the culture collection of Dr. Florent Trouillas (University of California, Davis). These isolates were originally recovered from symptomatic almond tissues and were identified to the subspecies/pathovar level through whole-genome sequencing followed by BLAST-based comparative analyses. Additional *Pseudomonas* strains infecting soybean, bean, tomato, cauliflower, radish, oleander, celery, tobacco, onion, and cherry, as well as soil-associated isolates, were obtained from the culture collections of Dr. Donald Cooksey via Dr. Caroline Roper (University of California, Riverside). These additional strains were included to assess cross-species infectivity (Table 1). Overnight bacterial cultures were grown in LB broth at 28 °C with shaking and adjusted to an OD600 of 2.0. For each strain, 100 µL of the standardized culture was mixed with 5 mL of molten LB top agar (0.5% agar) and poured onto LB agar plates to generate uniform bacterial lawns. High-titer phage suspensions (~ 10⁸ PFU/mL) were spotted (15 µL per spot) onto the lawns. Each phage-host combination was tested using three technical replicates, and the entire assay was repeated using three independent biological replicates conducted on separate days. Plates were incubated at 28 °C for 18–24 h before scoring. Phage infection was defined by the reproducible formation of visible lysis zones at the spot site. Clear or turbid lysis zones were recorded as positive interactions, whereas the absence of visible lysis was recorded as resistance.

To quantitatively validate host susceptibility, efficiency of plating (EOP) was determined for strains that produced lysis in spot assays. For each phage-host pair, phage stocks were serially 10-fold diluted in SM buffer, and 100 µL of each dilution was plated using the double-layer agar method as described above (100 µL host culture in 5 mL top agar). Plates were incubated at 28 °C for 18–24 h, and plaque-forming units (PFU/mL) were calculated from countable dilutions. EOP was defined as the ratio of PFU/mL on the test strain to PFU/mL on the phage propagation host (primary isolation host) assayed in parallel on the same day. EOP values were averaged across technical replicates within each experiment, and the assay was repeated across three independent biological replicates.

### Transmission electron microscopy

The morphology of phages vB_PsyP_Mobley, vB_PsyP_Plaza, and vB_PsyP_Mission was examined using a Talos L120C transmission electron microscope (Thermo Fisher Scientific) operated at 100 kV. High-titer phage lysates (~ 10⁹ PFU/mL) were prepared from freshly amplified stocks by centrifugation at 10,000 × g for 10 min to remove bacterial debris, followed by filtration through 0.22 μm syringe filters. For grid preparation, 10 µL of each phage suspension was applied to 400-mesh carbon-coated Formvar copper grids and allowed to adsorb for 2 min. Excess liquid was removed with filter paper, and grids were negatively stained with 2% (wt/vol) uranyl acetate for 30 s. After air-drying, grids were briefly rinsed with sterile distilled water to remove excess stain. At least two grids per phage were prepared and imaged independently. Morphological parameters, including capsid diameter and tail length, were measured using ImageJ software (v 1.40) from at least three intact virions per grid (≥ 6 virions per phage). Representative micrographs were captured at multiple magnifications to document structural features.

### Multiplicity of infection

The optimal multiplicity of infection (MOI) for phages vB_PsyP_Mobley, vB_PsyP_Plaza, and vB_PsyP_Mission was determined following standard protocols with minor modifications. MOI optimization was conducted using *Pseudomonas syringae* pv. *syringae* strain 2057, which served as the isolation and propagation host for all three phages, providing a standardized and biologically relevant reference for comparing phage infection dynamics. Overnight bacterial cultures were grown in LB broth at 28 °C with shaking (200 rpm) and adjusted to an OD₆₀₀ of 0.2 (approximately 10⁸ CFU/mL). High-titer phage suspensions (≥ 10⁹ PFU/mL) were prepared, and phage–bacterium mixtures were established across a range of MOIs (10, 1, 10^− 1^, 10^− 2^, 10^− 3^, and 10^− 4^). Each 500 µL mixture was incubated in LB broth at 28 °C with shaking for 5 h to allow phage replication. Following incubation, cultures were centrifuged (10,000 × g for 5 min), and supernatants were filtered through 0.22 μm syringe filters to remove residual bacterial cells. Phage titers were quantified using the double-layer agar method, in which 100 µL of serially diluted phage suspension was mixed with 100 µL of mid-log-phase bacterial culture in 0.5% LB soft agar and overlaid onto LB agar plates. Plaques were enumerated after overnight incubation at 28 °C. The MOI yielding the highest phage titer (PFU/mL) was defined as the optimal MOI for each phage. All assays were performed in triplicate and repeated on at least two independent occasions to ensure reproducibility.

### Thermal and pH viability assay

The stability of phages vB_PsyP_Mobley, vB_PsyP_Plaza, and vB_PsyP_Mission was evaluated across a range of temperature and pH conditions. For thermal stability assays, high-titer phage suspensions (~ 10⁸ PFU/mL) were aliquoted into sterile 1.5-mL microcentrifuge tubes and incubated for 24 h at 4, 25, 37, 50, or 60 °C using calibrated temperature-controlled incubators (refrigerated incubator for 4 °C, benchtop incubator for 25 and 37 °C, and dry heat block for 50 and 60 °C). For pH stability assays, phage suspensions were diluted into SM buffer adjusted to pH 1, 3, 5, 7, 9, 11, and 13 using 1 M HCl or NaOH and incubated at room temperature for 24 h. Following incubation, samples were briefly centrifuged to remove precipitates, and residual phage infectivity was quantified using the double-layer agar method. Briefly, 100 µL of serially diluted phage suspension was mixed with 100 µL of mid-log-phase *Pseudomonas syringae* culture in 0.5% LB soft agar and overlaid onto LB agar plates. Plates were incubated at 28 °C for 18–24 h, after which plaques were enumerated to determine phage titers (PFU/mL). Phage survival under each condition was expressed as a percentage of the initial titer measured at time zero. All assays were performed in triplicate and repeated in at least two independent experiments, and results were averaged across replicates.

### Phage adsorption

Adsorption kinetics of phages vB_PsyP_Mobley, vB_PsyP_Plaza, and vB_PsyP_Mission were determined using their previously established optimal MOIs and a standardized culture of *P. syringae* pv. *syringae*, following established methods with minor modifications^53^. Exponentially growing bacterial cultures (OD600 ≈ 0.2; ~10⁸ CFU/mL) were mixed with each phage at the optimal MOI and incubated at 28 °C with gentle shaking. For adsorption assays, 100 µL aliquots were withdrawn at 2-min intervals over a 20-min period and immediately centrifuged (12,000 × g, 1 min) to pellet bacterial cells. Supernatants containing unadsorbed phages were serially diluted and quantified using the double-layer agar method. The proportion of unadsorbed phages at each time point was calculated relative to titers at time zero, and adsorption constants were estimated by linear regression. All assays were performed in triplicate and repeated in at least two independent experiments to ensure reproducibility.

### Phage DNA extraction, sequencing, and genome assembly

Genomic DNA from purified phage particles was extracted using the Phage DNA Isolation Kit (Norgen Biotek, Thorold, Canada), following the manufacturer’s protocol. DNA concentration and purity were quantified using the Qubit Fluorometric Quantification System (Life Technologies, Carlsbad, CA, USA). Sequencing libraries were prepared and sequenced on the Illumina MiSeq platform using the MiSeq Reagent Kit v2 (500-cycle), generating paired-end reads of 250 base pairs^54^. To remove the adapters, N bases, and low-quality reads, the raw data were filtered using the Cutadapt program (http://code.google.com/p/cutadapt/) with the following parameters: −q 20 − m 20 -M 140. Quality of the processed reads was assessed with FastQC v0.11.7 (https://www.bioinformatics.babraham.ac.uk/projects/fastqc/). De novo assembly was performed using SPAdes v3.15.5 with k-mer sizes of 21, 33, and 55^55^. Low-coverage contigs were discarded to retain a high-confidence assembly. Assembly quality and completeness were evaluated using QUAST v5.1.0^56^ and CheckV vs. 1.0.1^57–59^. Genome termini were analyzed with PhageTerm to determine genome packaging and structure^48^. Functional annotation of the assembled genome was conducted using Pharokka v1.3.0^60^, with further manual curation. Protein-coding genes were predicted using PHANOTATE v1.5.1^61^, and tRNA genes were identified with tRNAscan-SE v2.0.11^62^. Putative depolymerase genes were identified using the DePolymerase Predictor (DePP) tool^63^. Comparative genomic analyses were performed using BLASTn against the NCBI nt database to identify the closest reference phages. Pairwise nucleotide identity was calculated using the virus intergenomic distance calculator (VIRIDIC)^64^ and genomic similarity matrices were generated accordingly. Whole genome synteny among the phages and their closest relatives was examined using clinker and clustermap.js^65^. The complete genome sequences of the isolated phages are available in GenBank under accession numbers **PX694326**,** PX694328**, and **PX694327**, respectively.

### Comparative genomics, phylogenomic, and taxonomic analyses

Comparative genomic analyses were performed using BLASTn searches against the NCBI nucleotide database to identify closely related reference phages. Pairwise nucleotide identity values were calculated using the Virus Intergenomic Distance Calculator (VIRIDIC), and similarity matrices were generated accordingly. Whole-genome comparisons and visualization of shared and unique genomic regions were conducted using BRIG and clinker with clustermap.js to assess gene content conservation and synteny among the three phages and their closest relatives. Reciprocal BLASTP analyses were performed to identify orthologous proteins shared between phage pairs and to quantify amino acid identity among structural, replication, and lysis-associated proteins. Phylogenomic relationships were further evaluated using ViPTree to place the phages within a broader viral proteomic context. To strengthen taxonomic inference, marker-gene phylogenies were constructed using the major capsid protein (MCP) and terminase large subunit (TerL). Homologous MCP and TerL sequences from closely related phages were retrieved from GenBank based on BLASTP searches. Protein sequences were aligned using MAFFT^66^, and maximum-likelihood phylogenetic trees were inferred using IQ-TREE (vs. 2.2.2.6) with automatic model selection and ultrafast bootstrap and SH-aLRT support^67^. Taxonomic classification was assigned according to current ICTV taxonomy based on genome-wide nucleotide identity, phylogenomic clustering, and marker-gene phylogenies. These combined analyses were used to evaluate genus- and species-level placement of the three phages within Autographiviridae.

### Phage lytic activity against planktonic and biofilm cells

The bactericidal activity of phages vB_PsyP_Mobley, vB_PsyP_Plaza, and vB_PsyP_Mission against planktonic *Pss* was assessed using a microplate-based growth inhibition assay. Overnight bacterial cultures were grown in LB broth at 28 °C with shaking (200 rpm), adjusted to an OD₆₀₀ of 0.2 (~ 10⁸ CFU/mL), and diluted 1:100 in fresh LB medium. Phage suspensions were prepared at high titer (~ 10⁸ PFU/mL), and bacteria were mixed with phages at varying MOIs (10, 1, 10^− 1^, 10^− 2^, 10^− 3^, and 10^− 4^). A total volume of 200 µL per well was dispensed into sterile, flat-bottom 96-well microtiter plates. Control wells included bacteria-only (growth control), phage-only (sterility control), and media-only (blank). Plates were incubated at 28 °C in a SpectraMax iD3 microplate reader (Molecular Devices, San Jose, CA, USA) with continuous orbital shaking. Optical density at 600 nm (OD₆₀₀) was recorded every 30 min over a 24 h period. Each treatment was performed in triplicate and repeated in at least two independent experiments. Biofilm inhibition was evaluated using a crystal violet (CV) staining assay in 96-well microtiter plates. *P. syringae* cultures were grown in LB medium, adjusted to an OD₆₀₀ of 0.8, and 100 µL aliquots were added to each well. Plates were incubated at 28 °C under static conditions for 24 h to allow biofilm establishment. Phages were then added at varying MOIs, and plates were incubated for an additional 24 h. Following incubation, wells were gently washed three times with sterile PBS to remove planktonic cells, stained with 0.1% (w/v) crystal violet for 20 min, rinsed thoroughly, and air-dried. Bound dye was solubilized in 95% ethanol, and absorbance was measured at 595 nm^68^ using the SpectraMax iD3 microplate reader. Biofilm biomass reduction was expressed relative to untreated controls. All treatments were conducted in triplicate, and assays were repeated independently at least twice.

### Statistical analysis

All experiments were performed with three technical replicates and repeated in at least two independent biological experiments unless otherwise stated. Data are presented as mean ± standard deviation (SD). Statistical analyses were conducted using GraphPad Prism (version 9.3.1). Depending on the experimental design, differences among groups were evaluated using one-way or two-way analysis of variance (ANOVA), followed by Tukey’s multiple comparisons test. Statistical significance was determined at *p* < 0.05, and significant differences among treatments are indicated by different lowercase letters in the figures.

## Supplementary Information

Below is the link to the electronic supplementary material.

Supplementary Material 1

Supplementary Material 2

Supplementary Material 3

Supplementary Material 4

Supplementary Material 5

## Data Availability

The complete genome sequences of phages vB_PsyP_Mobley, vB_PsyP_Mission, and vB_PsyP_Plaza are available in GenBank (NCBI) under accession numbers PX694326, PX694328, and PX694327, respectively.
