# Supplementary material for: Genomic and biological characterization of lytic phages infecting Pseudomonas syringae associated with almond bacterial blast
Source: Sci Rep. 2026 Apr 7;16:11657. doi: 10.1038/s41598-026-47496-5 (PMC13061930; doi:10.1038/s41598-026-47496-5)
Supplement: Supplementary file 1 — Supplementary Material 1 [file 41598_2026_47496_MOESM1_ESM.docx]

**Table S1:** Functional prediction of the phage vB_PsyP_Mobley, with supporting evidence

| **GENE** | **START** |  | **STOP** | | **FRAME** | **MMSEQS_EVAL** | **ANNOTATION** | **CATEGORY** |
| --- | --- | --- | --- | --- | --- | --- | --- | --- |
| LVUFTAYT_0001 | 1 |  | 1320 | + | | 2.62E-18 | tail fiber assembly | tail |
| LVUFTAYT_0002 | 1320 |  | 1604 | + | | 1.92E-08 | tail fiber protein | tail |
| LVUFTAYT_0003 | 1606 |  | 1812 | + | | 3.61E-06 | holin | lysis |
| LVUFTAYT_0004 | 1799 |  | 2110 | + | | 4.95E-18 | terminase small subunit | head and packaging |
| LVUFTAYT_0005 | 2107 |  | 3861 | + | | 1.02E-100 | terminase large subunit | head and packaging |
| LVUFTAYT_0006 | 3861 |  | 4073 | + | | No_PHROG | hypothetical protein | unknown function |
| LVUFTAYT_0007 | 4060 |  | 4266 | + | | 4.20E-08 | hypothetical protein | unknown function |
| LVUFTAYT_0008 | 4276 |  | 5670 | + | | 7.45E-14 | lipase | moron, auxiliary metabolic gene and host takeover |
| LVUFTAYT_0009 | 5679 |  | 6095 | + | | 4.91E-46 | endolysin | head and packaging |
| LVUFTAYT_0010 | 6092 |  | 6181 | + | | No_PHROG | hypothetical protein | unknown function |
| LVUFTAYT_0011 | 6182 |  | 6538 | + | | No_PHROG | hypothetical protein | unknown function |
| LVUFTAYT_0012 | 6538 |  | 6936 | + | | 7.77E-06 | Rz-like spanin | lysis |
| LVUFTAYT_0013 | 7027 |  | 7116 | + | | No_PHROG | hypothetical protein | unknown function |
| LVUFTAYT_0014 | 7488 |  | 7201 | - | | No_PHROG | hypothetical protein | unknown function |
| LVUFTAYT_0015 | 7786 |  | 7562 | - | | No_PHROG | hypothetical protein | unknown function |
| LVUFTAYT_0016 | 7882 |  | 7793 | - | | No_PHROG | hypothetical protein | unknown function |
| LVUFTAYT_0017 | 8111 |  | 7935 | - | | No_PHROG | hypothetical protein | unknown function |
| LVUFTAYT_0018 | 8133 |  | 8234 | + | | No_PHROG | hypothetical protein | unknown function |
| LVUFTAYT_0019 | 8303 |  | 8404 | + | | No_PHROG | hypothetical protein | unknown function |
| LVUFTAYT_0020 | 8462 |  | 8869 | + | | 1.92E-25 | hypothetical protein | unknown function |
| LVUFTAYT_0021 | 8866 |  | 9045 | + | | No_PHROG | hypothetical protein | unknown function |
| LVUFTAYT_0022 | 9052 |  | 9573 | + | | 1.19E-32 | hypothetical protein | unknown function |
| LVUFTAYT_0023 | 9626 |  | 10036 | + | | No_PHROG | hypothetical protein | unknown function |
| LVUFTAYT_0024 | 10068 |  | 10244 | + | | No_PHROG | hypothetical protein | unknown function |
| LVUFTAYT_0025 | 10288 |  | 10791 | + | | 2.46E-33 | hypothetical protein | unknown function |
| LVUFTAYT_0026 | 10863 |  | 11198 | + | | No_PHROG | hypothetical protein | unknown function |
| LVUFTAYT_0027 | 11268 |  | 11468 | + | | 3.36E-07 | hypothetical protein | unknown function |
| LVUFTAYT_0028 | 11465 |  | 11596 | + | | No_PHROG | hypothetical protein | unknown function |
| LVUFTAYT_0029 | 11696 |  | 12463 | + | | No_PHROG | hypothetical protein | unknown function |
| LVUFTAYT_0030 | 12460 |  | 12765 | + | | No_PHROG | hypothetical protein | unknown function |
| LVUFTAYT_0031 | 12740 |  | 13189 | + | | No_PHROG | hypothetical protein | unknown function |
| LVUFTAYT_0032 | 13284 |  | 13757 | + | | 2.87E-20 | hypothetical protein | unknown function |
| LVUFTAYT_0033 | 13770 |  | 14465 | + | | 5.92E-12 | 2OG-Fe(II) oxygenase | moron, auxiliary metabolic gene and host takeover |
| LVUFTAYT_0034 | 14465 |  | 15277 | + | | 1.68E-16 | DNA primase | DNA, RNA and nucleotide metabolism |
| LVUFTAYT_0035 | 15274 |  | 15651 | + | | No_PHROG | hypothetical protein | unknown function |
| LVUFTAYT_0036 | 15648 |  | 16931 | + | | 4.23E-30 | DnaB-like replicative helicase | DNA, RNA and nucleotide metabolism |
| LVUFTAYT_0037 | 16934 |  | 19261 | + | | 4.43E-130 | DNA polymerase | DNA, RNA and nucleotide metabolism |
| LVUFTAYT_0038 | 19273 |  | 20172 | + | | 3.19E-43 | hypothetical protein | unknown function |
| LVUFTAYT_0039 | 20226 |  | 21146 | + | | 8.99E-48 | exonuclease | DNA, RNA and nucleotide metabolism |
| LVUFTAYT_0040 | 21202 |  | 21351 | + | | No_PHROG | hypothetical protein | unknown function |
| LVUFTAYT_0041 | 21370 |  | 22341 | + | | 3.61E-99 | DNA polymerase exonuclease subunit | DNA, RNA and nucleotide metabolism |
| LVUFTAYT_0042 | 22338 |  | 22865 | + | | 3.74E-26 | hypothetical protein | unknown function |
| LVUFTAYT_0043 | 22862 |  | 23170 | + | | No_PHROG | hypothetical protein | unknown function |
| LVUFTAYT_0044 | 23167 |  | 23304 | + | | No_PHROG | hypothetical protein | unknown function |
| LVUFTAYT_0045 | 23306 |  | 24199 | + | | 8.82E-19 | ATP-dependent DNA ligase | DNA, RNA and nucleotide metabolism |
| LVUFTAYT_0046 | 24202 |  | 26637 | + | | 3.32E-91 | RNA polymerase | DNA, RNA and nucleotide metabolism |
| LVUFTAYT_0047 | 26688 |  | 26978 | + | | 5.22E-06 | hypothetical protein | unknown function |
| LVUFTAYT_0048 | 26975 |  | 27442 | + | | 6.85E-24 | hypothetical protein | unknown function |
| LVUFTAYT_0049 | 27435 |  | 27944 | + | | 6.13E-09 | hypothetical protein | unknown function |
| LVUFTAYT_0050 | 27956 |  | 29452 | + | | 5.73E-131 | head-tail adaptor | connector |
| LVUFTAYT_0051 | 29449 |  | 30177 | + | | 2.02E-27 | head scaffolding protein | head and packaging |
| LVUFTAYT_0052 | 30252 |  | 31253 | + | | 9.01E-40 | major head protein | head and packaging |
| LVUFTAYT_0053 | 31328 |  | 31927 | + | | 1.56E-46 | tail protein | tail |
| LVUFTAYT_0054 | 31915 |  | 34500 | + | | 9.59E-136 | tail protein | tail |
| LVUFTAYT_0055 | 34500 |  | 35222 | + | | 1.32E-57 | hypothetical protein | unknown function |
| LVUFTAYT_0056 | 35231 |  | 37474 | + | | 2.59E-80 | internal virion protein | head and packaging |
| LVUFTAYT_0057 | 37484 |  | 41188 | + | | 8.02E-78 | internal virion protein with endolysin domain | head and packaging |
| LVUFTAYT_0058 | 41244 |  | 41879 | + | | 2.20E-45 | tail fiber assembly | tail |

**Table S2:** Functional prediction of the phage vB_PsyP_Plaza, with supporting evidence

| **GENE** | **START** | **STOP** | | **FRAME** | **MMSEQS_EVAL** | **ANNOTATION** | **CATEGORY** |
| --- | --- | --- | --- | --- | --- | --- | --- |
| ZPHJMLSH_0001 | 58 | 255 | + | | No_PHROG | hypothetical protein | unknown function |
| ZPHJMLSH_0002 | 374 | 499 | + | | No_PHROG | hypothetical protein | unknown function |
| ZPHJMLSH_0003 | 737 | 874 | + | | No_PHROG | hypothetical protein | unknown function |
| ZPHJMLSH_0004 | 871 | 1125 | + | | 1.75E-55 | hypothetical protein | unknown function |
| ZPHJMLSH_0005 | 1125 | 1352 | + | | 7.77E-15 | hypothetical protein | unknown function |
| ZPHJMLSH_0006 | 1349 | 1591 | + | | 8.07E-49 | hypothetical protein | unknown function |
| ZPHJMLSH_0007 | 1681 | 2160 | + | | 3.27E-85 | hypothetical protein | unknown function |
| ZPHJMLSH_0008 | 2191 | 2733 | + | | 1.63E-81 | hypothetical protein | unknown function |
| ZPHJMLSH_0009 | 2730 | 3434 | + | | 1.83E-14 | 2OG-Fe(II) oxygenase | moron, auxiliary metabolic gene and host takeover |
| ZPHJMLSH_0010 | 3434 | 3694 | + | | 1.27E-15 | hypothetical protein | unknown function |
| ZPHJMLSH_0011 | 3696 | 4136 | + | | 7.22E-64 | hypothetical protein | unknown function |
| ZPHJMLSH_0012 | 4265 | 6922 | + | | 1.26E-126 | RNA polymerase | DNA, RNA and nucleotide metabolism |
| ZPHJMLSH_0013 | 6936 | 7073 | + | | 7.10E-35 | RNA polymerase | DNA, RNA and nucleotide metabolism |
| ZPHJMLSH_0014 | 7070 | 7405 | + | | 8.31E-08 | hypothetical protein | unknown function |
| ZPHJMLSH_0015 | 7405 | 7791 | + | | 3.34E-80 | hypothetical protein | unknown function |
| ZPHJMLSH_0016 | 7803 | 8867 | + | | 2.66E-43 | DNA ligase | DNA, RNA and nucleotide metabolism |
| ZPHJMLSH_0017 | 9060 | 9317 | + | | 2.58E-28 | hypothetical protein | unknown function |
| ZPHJMLSH_0018 | 9314 | 9961 | + | | 6.30E-23 | deoxynucleoside monophosphate kinase | other |
| ZPHJMLSH_0019 | 9958 | 10125 | + | | 7.60E-17 | RNA polymerase inhibitor | DNA, RNA and nucleotide metabolism |
| ZPHJMLSH_0020 | 10122 | 10487 | + | | 5.34E-75 | hypothetical protein | unknown function |
| ZPHJMLSH_0021 | 10541 | 11242 | + | | 6.81E-26 | Gp2.5-like ssDNA binding protein and ssDNA annealing protein | DNA, RNA and nucleotide metabolism |
| ZPHJMLSH_0022 | 11242 | 11685 | + | | 2.02E-73 | endonuclease | DNA, RNA and nucleotide metabolism |
| ZPHJMLSH_0023 | 11688 | 12128 | + | | 8.21E-69 | amidase | lysis |
| ZPHJMLSH_0024 | 12198 | 12737 | + | | 1.64E-52 | nucleotidyltransferase | DNA, RNA and nucleotide metabolism |
| ZPHJMLSH_0025 | 12724 | 14415 | + | | 1.43E-77 | DNA primase/helicase | DNA, RNA and nucleotide metabolism |
| ZPHJMLSH_0026 | 14434 | 14637 | + | | 1.99E-34 | hypothetical protein | unknown function |
| ZPHJMLSH_0027 | 14701 | 15210 | + | | 1.07E-55 | hypothetical protein | unknown function |
| ZPHJMLSH_0028 | 15221 | 17368 | + | | 1.40E-114 | DNA polymerase I | DNA, RNA and nucleotide metabolism |
| ZPHJMLSH_0029 | 17379 | 17762 | + | | 1.20E-14 | Gp5.5-like host HNS inhibition | moron, auxiliary metabolic gene and host takeover |
| ZPHJMLSH_0030 | 17755 | 17964 | + | | 6.86E-35 | hypothetical protein | unknown function |
| ZPHJMLSH_0031 | 17961 | 18905 | + | | 9.01E-35 | exonuclease | DNA, RNA and nucleotide metabolism |
| ZPHJMLSH_0032 | 18974 | 19216 | + | | 4.27E-38 | hypothetical protein | unknown function |
| ZPHJMLSH_0033 | 19219 | 19491 | + | | 2.39E-19 | hypothetical protein | unknown function |
| ZPHJMLSH_0034 | 19569 | 19931 | + | | 2.81E-50 | hypothetical protein | unknown function |
| ZPHJMLSH_0035 | 19903 | 20205 | + | | 1.06E-20 | host range and adsorption protein | moron, auxiliary metabolic gene and host takeover |
| ZPHJMLSH_0036 | 20220 | 21851 | + | | 1.48E-173 | head-tail adaptor | connector |
| ZPHJMLSH_0037 | 21920 | 22795 | + | | 2.35E-88 | head assembly | head and packaging |
| ZPHJMLSH_0038 | 22895 | 23938 | + | | 1.49E-120 | major head protein | head and packaging |
| ZPHJMLSH_0039 | 24002 | 24589 | + | | 1.67E-76 | tail protein | tail |
| ZPHJMLSH_0040 | 24599 | 27025 | + | | 3.95E-205 | tail protein | tail |
| ZPHJMLSH_0041 | 27084 | 27518 | + | | 7.65E-50 | internal virion protein | head and packaging |
| ZPHJMLSH_0042 | 27529 | 28110 | + | | 1.21E-48 | internal virion protein | head and packaging |
| ZPHJMLSH_0043 | 28110 | 30326 | + | | 3.76E-278 | internal virion protein | head and packaging |
| ZPHJMLSH_0044 | 30339 | 34517 | + | | 2.59E-306 | internal virion protein with endolysin domain | head and packaging |
| ZPHJMLSH_0045 | 34580 | 36463 | + | | 4.91E-225 | tail fiber protein | tail |
| ZPHJMLSH_0046 | 36501 | 36860 | + | | 4.57E-28 | tail fiber protein | tail |
| ZPHJMLSH_0047 | 36860 | 37075 | + | | 1.24E-19 | holin | lysis |
| ZPHJMLSH_0048 | 37072 | 37329 | + | | 1.94E-39 | terminase small subunit | head and packaging |
| ZPHJMLSH_0049 | 37329 | 37778 | + | | 1.58E-29 | Rz-like spanin | lysis |
| ZPHJMLSH_0050 | 37778 | 39526 | + | | 2.57E-103 | terminase large subunit | head and packaging |
| ZPHJMLSH_0051 | 39492 | 39602 | + | | No_PHROG | hypothetical protein | unknown function |
| ZPHJMLSH_0052 | 39705 | 39878 | + | | 3.53E-19 | hypothetical protein | unknown function |
| ZPHJMLSH_0053 | 39962 | 40078 | + | | No_PHROG | hypothetical protein | unknown function |

**Table S3:** Functional prediction of the phage vB_PsyP_Mission, with supporting evidence

| **GENE** | **START** | **STOP** | **FRAME** | **MMSEQS_EVAL** | **ANNOTATION** | **CATEGORY** |
| --- | --- | --- | --- | --- | --- | --- |
| BIMCHKFV_0001 | 3 | 422 | + | 6.11E-71 | DNA ligase | DNA, RNA and nucleotide metabolism |
| BIMCHKFV_0002 | 483 | 599 | + | No_PHROG | hypothetical protein | unknown function |
| BIMCHKFV_0003 | 666 | 1346 | + | 1.92E-28 | deoxynucleoside monophosphate kinase | other |
| BIMCHKFV_0004 | 1343 | 1501 | + | 3.23E-14 | RNA polymerase inhibitor | DNA, RNA and nucleotide metabolism |
| BIMCHKFV_0005 | 1498 | 1869 | + | 2.24E-69 | hypothetical protein | unknown function |
| BIMCHKFV_0006 | 1943 | 2635 | + | 1.26E-25 | Gp2.5-like ssDNA binding protein and ssDNA annealing protein | DNA, RNA and nucleotide metabolism |
| BIMCHKFV_0007 | 2638 | 3075 | + | 2.60E-69 | endonuclease | DNA, RNA and nucleotide metabolism |
| BIMCHKFV_0008 | 3087 | 3548 | + | 1.40E-64 | amidase | lysis |
| BIMCHKFV_0009 | 3615 | 4151 | + | 2.11E-42 | nucleotidyltransferase | DNA, RNA and nucleotide metabolism |
| BIMCHKFV_0010 | 4155 | 5846 | + | 1.41E-79 | DNA primase/helicase | DNA, RNA and nucleotide metabolism |
| BIMCHKFV_0011 | 5849 | 6067 | + | 1.04E-30 | hypothetical protein | unknown function |
| BIMCHKFV_0012 | 6126 | 6566 | + | 6.03E-84 | hypothetical protein | unknown function |
| BIMCHKFV_0013 | 6553 | 8691 | + | 5.68E-107 | DNA polymerase I | DNA, RNA and nucleotide metabolism |
| BIMCHKFV_0014 | 8691 | 9005 | + | 1.32E-19 | Gp5.5-like host HNS inhibition | moron, auxiliary metabolic gene and host takeover |
| BIMCHKFV_0015 | 9008 | 9217 | + | 4.28E-37 | hypothetical protein | unknown function |
| BIMCHKFV_0016 | 9214 | 10128 | + | 1.57E-40 | exonuclease | DNA, RNA and nucleotide metabolism |
| BIMCHKFV_0017 | 10887 | 10207 | - | No_PHROG | hypothetical protein | unknown function |
| BIMCHKFV_0018 | 10790 | 11107 | + | 1.23E-18 | host range and adsorption protein | moron, auxiliary metabolic gene and host takeover |
| BIMCHKFV_0019 | 11120 | 12727 | + | 1.64E-177 | head-tail adaptor | connector |
| BIMCHKFV_0020 | 12793 | 13704 | + | 1.38E-92 | head assembly | head and packaging |
| BIMCHKFV_0021 | 13793 | 14818 | + | 5.83E-112 | major head protein | head and packaging |
| BIMCHKFV_0022 | 14860 | 16233 | + | 7.53E-13 | structural protein with Ig domain | head and packaging |
| BIMCHKFV_0023 | 16300 | 16887 | + | 1.50E-75 | tail protein | tail |
| BIMCHKFV_0024 | 16898 | 19318 | + | 2.22E-203 | tail protein | tail |
| BIMCHKFV_0025 | 19348 | 19785 | + | 1.96E-49 | internal virion protein | head and packaging |
| BIMCHKFV_0026 | 19797 | 20354 | + | 1.84E-46 | internal virion protein | head and packaging |
| BIMCHKFV_0027 | 20363 | 22558 | + | 9.64E-296 | internal virion protein | head and packaging |
| BIMCHKFV_0028 | 22580 | 26575 | + | 0 | internal virion protein with endolysin domain | head and packaging |
| BIMCHKFV_0029 | 26633 | 28420 | + | 4.24E-203 | tail fiber protein | tail |
| BIMCHKFV_0030 | 28430 | 28621 | + | 6.53E-20 | holin | lysis |
| BIMCHKFV_0031 | 28627 | 28881 | + | 2.05E-36 | terminase small subunit | head and packaging |
| BIMCHKFV_0032 | 28881 | 29318 | + | 4.59E-33 | Rz-like spanin | lysis |
| BIMCHKFV_0033 | 29315 | 31072 | + | 1.46E-75 | terminase large subunit | head and packaging |
| BIMCHKFV_0034 | 31293 | 31466 | + | 4.40E-22 | hypothetical protein | unknown function |
| BIMCHKFV_0035 | 31552 | 31650 | + | No_PHROG | hypothetical protein | unknown function |
| BIMCHKFV_0036 | 31764 | 31862 | + | No_PHROG | hypothetical protein | unknown function |
| BIMCHKFV_0037 | 32021 | 32110 | + | No_PHROG | hypothetical protein | unknown function |
| BIMCHKFV_0038 | 32133 | 32234 | + | No_PHROG | hypothetical protein | unknown function |
| BIMCHKFV_0039 | 32269 | 32457 | + | No_PHROG | hypothetical protein | unknown function |
| BIMCHKFV_0040 | 32510 | 32992 | + | 9.45E-93 | hypothetical protein | unknown function |
| BIMCHKFV_0041 | 32992 | 33195 | + | 1.04E-30 | hypothetical protein | unknown function |
| BIMCHKFV_0042 | 33185 | 33307 | + | No_PHROG | hypothetical protein | unknown function |
| BIMCHKFV_0043 | 33320 | 33595 | + | 3.27E-48 | virion structural protein | head and packaging |
| BIMCHKFV_0044 | 33579 | 33905 | + | 4.80E-68 | hypothetical protein | unknown function |
| BIMCHKFV_0045 | 33966 | 34502 | + | 1.78E-82 | hypothetical protein | unknown function |
| BIMCHKFV_0046 | 34499 | 35203 | + | 1.67E-12 | 2OG-Fe(II) oxygenase | moron, auxiliary metabolic gene and host takeover |
| BIMCHKFV_0047 | 35206 | 35547 | + | 2.78E-36 | hypothetical protein | unknown function |
| BIMCHKFV_0048 | 35796 | 38321 | + | 3.82E-118 | RNA polymerase | DNA, RNA and nucleotide metabolism |
| BIMCHKFV_0049 | 38385 | 38576 | + | 2.24E-17 | hypothetical protein | unknown function |
| BIMCHKFV_0050 | 38573 | 38845 | + | 6.93E-55 | hypothetical protein | unknown function |
| BIMCHKFV_0051 | 38845 | 39828 | + | 2.25E-28 | DNA ligase | DNA, RNA and nucleotide metabolism |
