# Supplementary material for: Genomic and biological characterization of lytic phages infecting Pseudomonas syringae associated with almond bacterial blast
Source: Sci Rep. 2026 Apr 7;16:11657. doi: 10.1038/s41598-026-47496-5 (PMC13061930; doi:10.1038/s41598-026-47496-5)
Supplement: Supplementary file 2 — Supplementary Material 2 [file 41598_2026_47496_MOESM2_ESM.docx]

**Table S1:** Host range of vB_PsyP phages determined by plaque assays and efficiency of plating (EOP)

|  | **Host Strains** | **Isolates** | **Phylogroup**^1^ | **Bacterial host** | **Mobley** | | | **Plaza** | | | **Mission** | | | |
| --- | --- | --- | --- | --- | --- | --- | --- | --- | --- | --- | --- | --- | --- | --- |
| **1** | *Pss* | 288 | PG2 | Almond | 1.00E+09 | 1.20E+09 | 5.00E+08 | 9.00E+09 | 1.02E+10 | 9.90E+09 | 9.80E+09 | 1.35E+10 | 1.20E+10 |  |
| **2** | *Pss* | 2056 | PG2 | Almond | 7.30E+09 | 5.40E+09 | 6.60E+09 | 7.00E+08 | 1.20E+09 | 8.00E+08 | 1.40E+10 | 1.33E+10 | 1.45E+10 |  |
| **3** | *Pss* | 2057 | PG2 | Almond | 4.50E+09 | 4.80E+09 | 6.00E+09 | 5.00E+09 | 5.50E+09 | 7.10E+09 | 1.20E+10 | 1.05E+10 | 1.08E+10 |  |
| **4** | *Pss* | 2159 | PG2 | Almond | 4.10E+09 | 6.70E+09 | 5.50E+09 | 1.00E+10 | 1.03E+10 | 1.07E+10 | 1.20E+10 | 1.41E+10 | 1.28E+10 |  |
| **5** | *Pss* | 2219 | PG2 | Almond | 8.00E+08 | 3.00E+08 | 7.00E+08 | 5.00E+08 | 5.00E+08 | 6.00E+08 | 0.00E+00 | 0.00E+00 | 0.00E+00 |  |
| **6** | *Pss* | PS626 | PG2 | Almond | 2.00E+09 | 2.50E+09 | 3.10E+09 | 5.00E+08 | 1.20E+09 | 1.10E+09 | 1.10E+10 | 1.03E+10 | 1.15E+10 |  |
| **7** | *Pss* | PS631 | PG2 | Almond | 2.00E+09 | 2.50E+09 | 4.10E+09 | 0.00E+00 | 0.00E+00 | 0.00E+00 | 0.00E+00 | 0.00E+00 | 0.00E+00 |  |
| **8** | *Pss* | PS632 | PG2 | Almond | 1.10E+09 | 1.50E+09 | 1.00E+09 | 2.80E+09 | 3.10E+09 | 3.50E+09 | 1.60E+10 | 1.50E+10 | 1.48E+10 |  |
| **9** | *Pss* | PS685 | PG2 | Almond | 4.20E+09 | 3.90E+09 | 3.50E+09 | 9.00E+09 | 1.08E+10 | 9.70E+09 | 1.60E+10 | 1.48E+10 | 1.50E+10 |  |
| **10** | *Pss* | PS0693-10 | PG2 | Almond | 1.10E+09 | 1.30E+09 | 1.00E+09 | 8.00E+08 | 1.40E+09 | 7.00E+08 | 4.00E+09 | 3.20E+09 | 3.30E+09 |  |
| **11** | *Pss* | PS906 | PG2 | Almond | 2.80E+09 | 4.00E+09 | 3.30E+09 | 0.00E+00 | 0.00E+00 | 0.00E+00 | 1.30E+09 | 1.90E+09 | 2.30E+09 |  |
| **12** | *Pss* | PS914 | PG2 | Almond | 1.20E+09 | 1.50E+09 | 1.80E+09 | 7.00E+08 | 7.00E+08 | 9.00E+08 | 1.05E+10 | 1.09E+10 | 8.90E+09 |  |
| **13** | *Pss* | PS926 | PG2 | Almond | 3.20E+09 | 2.80E+09 | 2.20E+09 | 5.00E+08 | 8.00E+08 | 1.20E+09 | 4.40E+09 | 5.00E+09 | 3.80E+09 |  |
| **14** | *Pss* | JEA288 | PG2 | Almond | 9.00E+08 | 1.00E+09 | 7.00E+08 | 1.00E+10 | 7.50E+09 | 8.00E+09 | 1.40E+10 | 1.30E+10 | 1.28E+10 |  |
| **15** | *P. viridiflava* | PS990 | PG7 | Almond | 0.00E+00 | 0.00E+00 | 0.00E+00 | 0.00E+00 | 0.00E+00 | 0.00E+00 | 1.00E+09 | 1.21E+10 | 1.11E+10 |  |
| **16** | *P. viridiflava* | PS991 | PG7 | Almond | 7.00E+08 | 1.10E+09 | 6.00E+08 | 1.20E+09 | 1.50E+09 | 8.00E+08 | 1.41E+10 | 1.45E+10 | 1.49E+10 |  |
| **17** | *P. viridiflava* | PS992 | PG7 | Almond | 0.00E+00 | 0.00E+00 | 0.00E+00 | 0.00E+00 | 0.00E+00 | 0.00E+00 | 1.00E+10 | 1.05E+10 | 8.90E+09 |  |
| **18** | *P. viridiflava* | PS993 | PG7 | Almond | 3.00E+08 | 5.00E+08 | 6.00E+08 | 5.00E+09 | 6.10E+09 | 6.50E+09 | 9.00E+09 | 1.00E+10 | 1.21E+10 |  |
| **19** | *P. viridiflava* | PS996 | PG7 | Almond | 1.30E+09 | 1.20E+09 | 1.10E+09 | 8.00E+08 | 1.00E+09 | 1.20E+09 | 1.10E+09 | 1.40E+09 | 9.00E+08 |  |
| **20** | *P. viridiflava* | PS998 | PG7 | Almond | 0.00E+00 | 0.00E+00 | 0.00E+00 | 0.00E+00 | 0.00E+00 | 0.00E+00 | 0.00E+00 | 0.00E+00 | 0.00E+00 |  |
| **21** | *P. viridiflava* | PS951 | PG7 | Almond | 0.00E+00 | 0.00E+00 | 0.00E+00 | 0.00E+00 | 0.00E+00 | 0.00E+00 | 1.51E+10 | 1.67E+10 | 1.66E+10 |  |
| **22** | *P. viridiflava* | PS1039 | PG7 | Almond | 1.10E+09 | 1.20E+09 | 1.30E+09 | 3.40E+09 | 3.50E+09 | 4.00E+09 | 1.50E+10 | 1.26E+10 | 1.34E+10 |  |
| **23** | *P. viridiflava* | PS1136 | PG7 | Almond | 0.00E+00 | 0.00E+00 | 0.00E+00 | 0.00E+00 | 0.00E+00 | 0.00E+00 | 3.00E+08 | 7.00E+08 | 1.10E+09 |  |
| 24 | P. viridiflava | PS1137 | PG7 | Almond | 0.00E+00 | 0.00E+00 | 0.00E+00 | 0.00E+00 | 0.00E+00 | 0.00E+00 | 1.00E+09 | 1.00E+09 | 1.10E+09 |  |
| **25** | *Ps* pv. *glycinea* | 0893-26 | N/A | Soybean | 0.00E+00 | 0.00E+00 | 0.00E+00 | 0.00E+00 | 0.00E+00 | 0.00E+00 | 0.00E+00 | 0.00E+00 | 0.00E+00 |  |
| **26** | *Ps* pv. *phaseolicola* | 0893-64 | N/A | Bean | 0.00E+00 | 0.00E+00 | 0.00E+00 | 0.00E+00 | 0.00E+00 | 0.00E+00 | 2.00E+09 | 2.70E+09 | 1.90E+09 |  |
| **27** | *Ps* pv. *tomato* DC3000 | 1097-18 | N/A | Tomato | 1.50E+09 | 1.50E+09 | 1.20E+09 | 1.80E+09 | 9.00E+08 | 1.20E+09 | 8.90E+09 | 7.00E+09 | 8.80E+09 |  |
| **28** | *P. maculicola* | 0190-2 | N/A | Cauliflower | 2.00E+09 | 2.10E+09 | 1.60E+09 | 2.80E+09 | 1.70E+09 | 2.20E+09 | 5.90E+09 | 5.50E+09 | 7.00E+09 |  |
| **29** | *Ps* pv. *savastanoi* | 0693-10 | N/A | Oleander | 0.00E+00 | 0.00E+00 | 0.00E+00 | 0.00E+00 | 0.00E+00 | 0.00E+00 | 1.20E+09 | 1.50E+09 | 9.00E+08 |  |
| **30** | *P. fluorescens* isolate C8.2 | 0990-6 | N/A | Tobacco | 0.00E+00 | 0.00E+00 | 0.00E+00 | 0.00E+00 | 0.00E+00 | 0.00E+00 | 0.00E+00 | 0.00E+00 | 0.00E+00 |  |
| **31** | *P. cepacia* 945 | 1282-2 | N/A | Onion | 0.00E+00 | 0.00E+00 | 0.00E+00 | 0.00E+00 | 0.00E+00 | 0.00E+00 | 0.00E+00 | 0.00E+00 | 0.00E+00 |  |
| **32** | *Ps* pv. *apii* | 0790-4 | N/A | Celery | 0.00E+00 | 0.00E+00 | 0.00E+00 | 0.00E+00 | 0.00E+00 | 0.00E+00 | 0.00E+00 | 0.00E+00 | 0.00E+00 |  |
| **33** | *P. corrugata* | 0682-12 | N/A | Tomato | 0.00E+00 | 0.00E+00 | 0.00E+00 | 0.00E+00 | 0.00E+00 | 0.00E+00 | 0.00E+00 | 0.00E+00 | 0.00E+00 |  |
| **34** | *P. putida* | 0882-9 | N/A | Soil | 0.00E+00 | 0.00E+00 | 0.00E+00 | 0.00E+00 | 0.00E+00 | 0.00E+00 | 0.00E+00 | 0.00E+00 | 0.00E+00 |  |
| **35** | *Ps* pv. *atropurpurea* | 0791-15 | N/A | Cherry | 0.00E+00 | 0.00E+00 | 0.00E+00 | 0.00E+00 | 0.00E+00 | 0.00E+00 | 0.00E+00 | 0.00E+00 | 0.00E+00 |  |
| **36** | *P. viridiflava* | 1294-15 | N/A | Radish | 0.00E+00 | 0.00E+00 | 0.00E+00 | 0.00E+00 | 0.00E+00 | 0.00E+00 | 1.50E+09 | 3.00E+09 | 2.80E+09 |  |

^1^ Phylogroup assignments are based on previous characterization of the isolates.

**Table S2:** Functional prediction of the phage vB_PsyP_Mobley, with supporting evidence

| **GENE** | **START** |  | **STOP** | | **FRAME** | **MMSEQS_EVAL** | **ANNOTATION** | **CATEGORY** |
| --- | --- | --- | --- | --- | --- | --- | --- | --- |
| UZVTFFVZ_CDS_0001 | 1658 | 3 | - | 4.44E-130 | | DNA polymerase | DNA, RNA and nucleotide metabolism | UZVTFFVZ_CDS_0001 |
| UZVTFFVZ_CDS_0002 | 2944 | 1661 | - | 4.23E-30 | | DnaB-like replicative helicase | DNA, RNA and nucleotide metabolism | UZVTFFVZ_CDS_0002 |
| UZVTFFVZ_CDS_0003 | 3318 | 2941 | - | No_PHROG | | hypothetical protein | unknown function | UZVTFFVZ_CDS_0003 |
| UZVTFFVZ_CDS_0004 | 4130 | 3315 | - | 1.69E-16 | | DNA primase | DNA, RNA and nucleotide metabolism | UZVTFFVZ_CDS_0004 |
| UZVTFFVZ_CDS_0005 | 4822 | 4127 | - | 5.93E-12 | | 2OG-Fe(II) oxygenase | moron, auxiliary metabolic gene and host takeover | UZVTFFVZ_CDS_0005 |
| UZVTFFVZ_CDS_0006 | 5308 | 4835 | - | 2.88E-20 | | hypothetical protein | unknown function | UZVTFFVZ_CDS_0006 |
| UZVTFFVZ_CDS_0007 | 5852 | 5403 | - | No_PHROG | | hypothetical protein | unknown function | UZVTFFVZ_CDS_0007 |
| UZVTFFVZ_CDS_0008 | 6132 | 5827 | - | No_PHROG | | hypothetical protein | unknown function | UZVTFFVZ_CDS_0008 |
| UZVTFFVZ_CDS_0009 | 6896 | 6129 | - | No_PHROG | | hypothetical protein | unknown function | UZVTFFVZ_CDS_0009 |
| UZVTFFVZ_CDS_0010 | 7127 | 6996 | - | No_PHROG | | hypothetical protein | unknown function | UZVTFFVZ_CDS_0010 |
| UZVTFFVZ_CDS_0011 | 7324 | 7124 | - | 3.36E-07 | | hypothetical protein | unknown function | UZVTFFVZ_CDS_0011 |
| UZVTFFVZ_CDS_0012 | 7729 | 7394 | - | No_PHROG | | hypothetical protein | unknown function | UZVTFFVZ_CDS_0012 |
| UZVTFFVZ_CDS_0013 | 8304 | 7801 | - | 2.47E-33 | | hypothetical protein | unknown function | UZVTFFVZ_CDS_0013 |
| UZVTFFVZ_CDS_0014 | 8524 | 8348 | - | No_PHROG | | hypothetical protein | unknown function | UZVTFFVZ_CDS_0014 |
| UZVTFFVZ_CDS_0015 | 8966 | 8556 | - | No_PHROG | | hypothetical protein | unknown function | UZVTFFVZ_CDS_0015 |
| UZVTFFVZ_CDS_0016 | 9540 | 9019 | - | 1.20E-32 | | hypothetical protein | unknown function | UZVTFFVZ_CDS_0016 |
| UZVTFFVZ_CDS_0017 | 9726 | 9547 | - | No_PHROG | | hypothetical protein | unknown function | UZVTFFVZ_CDS_0017 |
| UZVTFFVZ_CDS_0018 | 10130 | 9723 | - | 1.92E-25 | | hypothetical protein | unknown function | UZVTFFVZ_CDS_0018 |
| UZVTFFVZ_CDS_0019 | 10289 | 10188 | - | No_PHROG | | hypothetical protein | unknown function | UZVTFFVZ_CDS_0019 |
| UZVTFFVZ_CDS_0020 | 10459 | 10358 | - | No_PHROG | | hypothetical protein | unknown function | UZVTFFVZ_CDS_0020 |
| UZVTFFVZ_CDS_0021 | 10481 | 10657 | + | No_PHROG | | hypothetical protein | unknown function | UZVTFFVZ_CDS_0021 |
| UZVTFFVZ_CDS_0022 | 10710 | 10799 | + | No_PHROG | | hypothetical protein | unknown function | UZVTFFVZ_CDS_0022 |
| UZVTFFVZ_CDS_0023 | 10806 | 11030 | + | No_PHROG | | hypothetical protein | unknown function | UZVTFFVZ_CDS_0023 |
| UZVTFFVZ_CDS_0024 | 11104 | 11391 | + | No_PHROG | | hypothetical protein | unknown function | UZVTFFVZ_CDS_0024 |
| UZVTFFVZ_CDS_0025 | 11565 | 11476 | - | No_PHROG | | hypothetical protein | unknown function | UZVTFFVZ_CDS_0025 |
| UZVTFFVZ_CDS_0026 | 12054 | 11656 | - | 7.79E-06 | | Rz-like spanin | lysis | UZVTFFVZ_CDS_0026 |
| UZVTFFVZ_CDS_0027 | 12410 | 12054 | - | No_PHROG | | hypothetical protein | unknown function | UZVTFFVZ_CDS_0027 |
| UZVTFFVZ_CDS_0028 | 12500 | 12411 | - | No_PHROG | | hypothetical protein | unknown function | UZVTFFVZ_CDS_0028 |
| UZVTFFVZ_CDS_0029 | 12913 | 12497 | - | 4.92E-46 | | endolysin | head and packaging | UZVTFFVZ_CDS_0029 |
| UZVTFFVZ_CDS_0030 | 14316 | 12922 | - | 7.46E-14 | | lipase | moron, auxiliary metabolic gene and host takeover | UZVTFFVZ_CDS_0030 |
| UZVTFFVZ_CDS_0031 | 14532 | 14326 | - | 4.21E-08 | | hypothetical protein | unknown function | UZVTFFVZ_CDS_0031 |
| UZVTFFVZ_CDS_0032 | 14731 | 14519 | - | No_PHROG | | hypothetical protein | unknown function | UZVTFFVZ_CDS_0032 |
| UZVTFFVZ_CDS_0033 | 16485 | 14731 | - | 1.02E-100 | | terminase large subunit | head and packaging | UZVTFFVZ_CDS_0033 |
| UZVTFFVZ_CDS_0034 | 16793 | 16482 | - | 4.96E-18 | | terminase small subunit | head and packaging | UZVTFFVZ_CDS_0034 |
| UZVTFFVZ_CDS_0035 | 16986 | 16780 | - | 3.62E-06 | | holin | lysis | UZVTFFVZ_CDS_0035 |
| UZVTFFVZ_CDS_0036 | 17272 | 16988 | - | 1.92E-08 | | tail fiber protein | tail | UZVTFFVZ_CDS_0036 |
| UZVTFFVZ_CDS_0037 | 19227 | 17272 | - | 1.72E-69 | | tail fiber assembly | tail | UZVTFFVZ_CDS_0037 |
| UZVTFFVZ_CDS_0038 | 22987 | 19283 | - | 8.04E-78 | | internal virion protein with endolysin domain | head and packaging | UZVTFFVZ_CDS_0038 |
| UZVTFFVZ_CDS_0039 | 25240 | 22997 | - | 2.60E-80 | | internal virion protein | head and packaging | UZVTFFVZ_CDS_0039 |
| UZVTFFVZ_CDS_0040 | 25971 | 25249 | - | 1.32E-57 | | hypothetical protein | unknown function | UZVTFFVZ_CDS_0040 |
| UZVTFFVZ_CDS_0041 | 28544 | 25971 | - | 9.61E-136 | | tail protein | tail | UZVTFFVZ_CDS_0041 |
| UZVTFFVZ_CDS_0042 | 29143 | 28544 | - | 1.56E-46 | | tail protein | tail | UZVTFFVZ_CDS_0042 |
| UZVTFFVZ_CDS_0043 | 30219 | 29218 | - | 9.03E-40 | | major head protein | head and packaging | UZVTFFVZ_CDS_0043 |
| UZVTFFVZ_CDS_0044 | 31022 | 30294 | - | 2.02E-27 | | head scaffolding protein | head and packaging | UZVTFFVZ_CDS_0044 |
| UZVTFFVZ_CDS_0045 | 32515 | 31019 | - | 5.74E-131 | | head-tail adaptor | connector | UZVTFFVZ_CDS_0045 |
| UZVTFFVZ_CDS_0046 | 33036 | 32527 | - | 6.14E-09 | | hypothetical protein | unknown function | UZVTFFVZ_CDS_0046 |
| UZVTFFVZ_CDS_0047 | 33496 | 33029 | - | 6.86E-24 | | hypothetical protein | unknown function | UZVTFFVZ_CDS_0047 |
| UZVTFFVZ_CDS_0048 | 33783 | 33493 | - | 5.23E-06 | | hypothetical protein | unknown function | UZVTFFVZ_CDS_0048 |
| UZVTFFVZ_CDS_0049 | 36269 | 33834 | - | 3.33E-91 | | RNA polymerase | DNA, RNA and nucleotide metabolism | UZVTFFVZ_CDS_0049 |
| UZVTFFVZ_CDS_0050 | 37165 | 36272 | - | 8.84E-19 | | ATP-dependent DNA ligase | DNA, RNA and nucleotide metabolism | UZVTFFVZ_CDS_0050 |
| UZVTFFVZ_CDS_0051 | 37304 | 37167 | - | No_PHROG | | hypothetical protein | unknown function | UZVTFFVZ_CDS_0051 |
| UZVTFFVZ_CDS_0052 | 37609 | 37301 | - | No_PHROG | | hypothetical protein | unknown function | UZVTFFVZ_CDS_0052 |
| UZVTFFVZ_CDS_0053 | 38133 | 37606 | - | 3.74E-26 | | hypothetical protein | unknown function | UZVTFFVZ_CDS_0053 |
| UZVTFFVZ_CDS_0054 | 38954 | 38130 | - | 3.61E-99 | | DNA polymerase exonuclease subunit | DNA, RNA and nucleotide metabolism | UZVTFFVZ_CDS_0054 |
| UZVTFFVZ_CDS_0055 | 39337 | 38951 | - | 1.17E-35 | | endonuclease VII | DNA, RNA and nucleotide metabolism | UZVTFFVZ_CDS_0055 |
| UZVTFFVZ_CDS_0056 | 40239 | 39325 | - | 9.01E-48 | | exonuclease | DNA, RNA and nucleotide metabolism | UZVTFFVZ_CDS_0056 |
| UZVTFFVZ_CDS_0057 | 41198 | 40299 | - | 3.19E-43 | | hypothetical protein | unknown function | UZVTFFVZ_CDS_0057 |
| UZVTFFVZ_CDS_0058 | 41878 | 41210 | - | 5.38E-16 | | DNA polymerase | DNA, RNA and nucleotide metabolism | UZVTFFVZ_CDS_0058 |

**Table S3:** Functional prediction of the phage vB_PsyP_Plaza, with supporting evidence

| **GENE** | **START** | **STOP** | | **FRAME** | **MMSEQS_EVAL** | **ANNOTATION** | **CATEGORY** |
| --- | --- | --- | --- | --- | --- | --- | --- |
| UMWGZUHC_CDS_0001 | 462 | 1 | - | | 8.56E-41 | Tail protein | Tail |
| UMWGZUHC_CDS_0002 | 1059 | 472 | - | | 1.66E-76 | Tail protein | Tail |
| UMWGZUHC_CDS_0003 | 1959 | 1123 | - | | 1.27E-86 | Major head protein | Head and packaging |
| UMWGZUHC_CDS_0004 | 2192 | 2061 | - | | No_PHROG | Hypothetical protein | Unknown function |
| UMWGZUHC_CDS_0005 | 3100 | 2225 | - | | 2.35E-88 | Head assembly | Head and packaging |
| UMWGZUHC_CDS_0006 | 4800 | 3169 | - | | 1.48E-173 | Head-tail adaptor | Connector |
| UMWGZUHC_CDS_0007 | 5117 | 4815 | - | | 1.06E-20 | Host range and adsorption protein | Moron, auxiliary metabolic gene and host takeover |
| UMWGZUHC_CDS_0008 | 5451 | 5089 | - | | 2.80E-50 | Hypothetical protein | Unknown function |
| UMWGZUHC_CDS_0009 | 5801 | 5529 | - | | 2.39E-19 | Hypothetical protein | Unknown function |
| UMWGZUHC_CDS_0010 | 6046 | 5804 | - | | 4.26E-38 | Hypothetical protein | Unknown function |
| UMWGZUHC_CDS_0011 | 7059 | 6115 | - | | 8.99E-35 | Exonuclease | Dna, rna and nucleotide metabolism |
| UMWGZUHC_CDS_0012 | 7265 | 7056 | - | | 6.84E-35 | Hypothetical protein | Unknown function |
| UMWGZUHC_CDS_0013 | 7641 | 7258 | - | | 1.20E-14 | Gp5.5-like host HNS inhibition | Moron, auxiliary metabolic gene and host takeover |
| UMWGZUHC_CDS_0014 | 9799 | 7652 | - | | 1.39E-114 | DNA polymerase I | DNA, RNA and nucleotide metabolism |
| UMWGZUHC_CDS_0015 | 10319 | 9810 | - | | 1.06E-55 | Hypothetical protein | Unknown function |
| UMWGZUHC_CDS_0016 | 10586 | 10383 | - | | 1.99E-34 | Hypothetical protein | Unknown function |
| UMWGZUHC_CDS_0017 | 12296 | 10605 | - | | 1.42E-77 | DNA primase/helicase | DNA, RNA and nucleotide metabolism |
| UMWGZUHC_CDS_0018 | 12822 | 12283 | - | | 1.63E-52 | Nucleotidyltransferase | Dna, rna and nucleotide metabolism |
| UMWGZUHC_CDS_0019 | 13332 | 12892 | - | | 8.19E-69 | Amidase | Lysis |
| UMWGZUHC_CDS_0020 | 13778 | 13335 | - | | 2.02E-73 | Endonuclease | Dna, rna and nucleotide metabolism |
| UMWGZUHC_CDS_0021 | 14479 | 13778 | - | | 6.79E-26 | Gp2.5-like ssdna binding protein and ssdna annealing protein | DNA, RNA and nucleotide metabolism |
| UMWGZUHC_CDS_0022 | 14898 | 14533 | - | | 5.33E-75 | Hypothetical protein | Unknown function |
| UMWGZUHC_CDS_0023 | 15062 | 14895 | - | | 7.58E-17 | RNA polymerase inhibitor | DNA, RNA and nucleotide metabolism |
| UMWGZUHC_CDS_0024 | 15706 | 15059 | - | | 6.29E-23 | Deoxynucleoside monophosphate kinase | Other |
| UMWGZUHC_CDS_0025 | 15960 | 15703 | - | | 2.57E-28 | Hypothetical protein | Unknown function |
| UMWGZUHC_CDS_0026 | 17217 | 16153 | - | | 2.66E-43 | DNA ligase | DNA, RNA and nucleotide metabolism |
| UMWGZUHC_CDS_0027 | 17615 | 17229 | - | | 3.33E-80 | Hypothetical protein | Unknown function |
| UMWGZUHC_CDS_0028 | 17950 | 17615 | - | | 8.30E-08 | Hypothetical protein | Unknown function |
| UMWGZUHC_CDS_0029 | 18084 | 17947 | - | | 7.09E-35 | RNA polymerase | DNA, RNA and nucleotide metabolism |
| UMWGZUHC_CDS_0030 | 20755 | 18098 | - | | 1.26E-126 | RNA polymerase | DNA, RNA and nucleotide metabolism |
| UMWGZUHC_CDS_0031 | 21324 | 20884 | - | | 7.20E-64 | Hypothetical protein | Unknown function |
| UMWGZUHC_CDS_0032 | 21586 | 21326 | - | | 1.26E-15 | Hypothetical protein | Unknown function |
| UMWGZUHC_CDS_0033 | 22290 | 21586 | - | | 1.82E-14 | 2OG-Fe(II) oxygenase | Moron, auxiliary metabolic gene and host takeover |
| UMWGZUHC_CDS_0034 | 22829 | 22287 | - | | 1.63E-81 | Hypothetical protein | Unknown function |
| UMWGZUHC_CDS_0035 | 23339 | 22860 | - | | 3.26E-85 | Hypothetical protein | Unknown function |
| UMWGZUHC_CDS_0036 | 23671 | 23429 | - | | 8.06E-49 | Hypothetical protein | Unknown function |
| UMWGZUHC_CDS_0037 | 23895 | 23668 | - | | 7.75E-15 | Hypothetical protein | Unknown function |
| UMWGZUHC_CDS_0038 | 24149 | 23895 | - | | 1.74E-55 | Hypothetical protein | Unknown function |
| UMWGZUHC_CDS_0039 | 24283 | 24146 | - | | No_PHROG | Hypothetical protein | Unknown function |
| UMWGZUHC_CDS_0040 | 24646 | 24521 | - | | No_PHROG | Hypothetical protein | Unknown function |
| UMWGZUHC_CDS_0041 | 24962 | 24765 | - | | No_PHROG | Hypothetical protein | Unknown function |
| UMWGZUHC_CDS_0042 | 25198 | 25082 | - | | No_PHROG | Hypothetical protein | Unknown function |
| UMWGZUHC_CDS_0043 | 25455 | 25282 | - | | 3.52E-19 | Hypothetical protein | Unknown function |
| UMWGZUHC_CDS_0044 | 25671 | 25558 | - | | No_PHROG | Hypothetical protein | Unknown function |
| UMWGZUHC_CDS_0045 | 27382 | 25634 | - | | 2.56E-103 | Terminase large subunit | Head and packaging |
| UMWGZUHC_CDS_0046 | 27831 | 27382 | - | | 1.58E-29 | Rz-like spanin | Lysis |
| UMWGZUHC_CDS_0047 | 28088 | 27831 | - | | 1.94E-39 | Terminase small subunit | Head and packaging |
| UMWGZUHC_CDS_0048 | 28300 | 28085 | - | | 1.24E-19 | Holin | Lysis |
| UMWGZUHC_CDS_0049 | 28659 | 28300 | - | | 4.56E-28 | Tail fiber protein | Tail |
| UMWGZUHC_CDS_0050 | 30580 | 28697 | - | | 4.90E-225 | Tail fiber protein | Tail |
| UMWGZUHC_CDS_0051 | 34821 | 30643 | - | | 2.58E-306 | Internal virion protein with endolysin domain | Head and packaging |
| UMWGZUHC_CDS_0052 | 37050 | 34834 | - | | 3.75E-278 | Internal virion protein | Head and packaging |
| UMWGZUHC_CDS_0053 | 37631 | 37050 | - | | 1.21E-48 | Internal virion protein | Head and packaging |

| UMWGZUHC_CDS_0054 | 38076 | 37642 | - | 7.64E-50 | Internal virion protein | Head and packaging |
| --- | --- | --- | --- | --- | --- | --- |
| UMWGZUHC_CDS_0055 | 40099 | 38135 | - | 8.15E-165 | Tail protein | Tail |

**Table S4:** Functional prediction of the phage vB_PsyP_Mission, with supporting evidence

| **GENE** | **START** | **STOP** | **FRAME** | **MMSEQS_EVAL** | **ANNOTATION** | **CATEGORY** |
| --- | --- | --- | --- | --- | --- | --- |
| PJZEDJXZ_CDS_0001 | 1 | 1911 | + | 7.34E-165 | Internal virion protein with endolysin domain | Head and packaging |
| PJZEDJXZ_CDS_0002 | 1969 | 3756 | + | 4.25E-203 | Tail fiber protein | Tail |
| PJZEDJXZ_CDS_0003 | 3766 | 3957 | + | 6.54E-20 | Holin | Lysis |
| PJZEDJXZ_CDS_0004 | 3963 | 4217 | + | 2.05E-36 | Terminase small subunit | Head and packaging |
| PJZEDJXZ_CDS_0005 | 4217 | 4654 | + | 4.60E-33 | Rz-like spanin | Lysis |
| PJZEDJXZ_CDS_0006 | 4651 | 6408 | + | 1.46E-75 | Terminase large subunit | Head and packaging |
| PJZEDJXZ_CDS_0007 | 6629 | 6802 | + | 4.41E-22 | Hypothetical protein | Unknown function |
| PJZEDJXZ_CDS_0008 | 6888 | 6986 | + | No_PHROG | Hypothetical protein | Unknown function |
| PJZEDJXZ_CDS_0009 | 7100 | 7198 | + | No_PHROG | Hypothetical protein | Unknown function |
| PJZEDJXZ_CDS_0010 | 7357 | 7446 | + | No_PHROG | Hypothetical protein | Unknown function |
| PJZEDJXZ_CDS_0011 | 7469 | 7570 | + | No_PHROG | Hypothetical protein | Unknown function |
| PJZEDJXZ_CDS_0012 | 7605 | 7793 | + | No_PHROG | Hypothetical protein | Unknown function |
| PJZEDJXZ_CDS_0013 | 7846 | 8328 | + | 9.47E-93 | Hypothetical protein | Unknown function |
| PJZEDJXZ_CDS_0014 | 8328 | 8531 | + | 1.04E-30 | Hypothetical protein | Unknown function |
| PJZEDJXZ_CDS_0015 | 8521 | 8643 | + | No_PHROG | Hypothetical protein | Unknown function |
| PJZEDJXZ_CDS_0016 | 8656 | 8931 | + | 3.28E-48 | Virion structural protein | Head and packaging |
| PJZEDJXZ_CDS_0017 | 8915 | 9241 | + | 4.81E-68 | Hypothetical protein | Unknown function |
| PJZEDJXZ_CDS_0018 | 9302 | 9838 | + | 1.79E-82 | Hypothetical protein | Unknown function |
| PJZEDJXZ_CDS_0019 | 9835 | 10539 | + | 1.67E-12 | 2OG-Fe(II) oxygenase | Moron, auxiliary metabolic gene and host takeover |
| PJZEDJXZ_CDS_0020 | 10542 | 10883 | + | 2.79E-36 | Hypothetical protein | Unknown function |
| PJZEDJXZ_CDS_0021 | 11132 | 13657 | + | 3.83E-118 | RNA polymerase | DNA, RNA and nucleotide metabolism |
| PJZEDJXZ_CDS_0022 | 13721 | 13912 | + | 2.24E-17 | Hypothetical protein | Unknown function |
| PJZEDJXZ_CDS_0023 | 13909 | 14181 | + | 6.94E-55 | Hypothetical protein | Unknown function |
| PJZEDJXZ_CDS_0024 | 14181 | 15164 | + | 2.25E-28 | DNA ligase | DNA, RNA and nucleotide metabolism |
| PJZEDJXZ_CDS_0025 | 15241 | 15687 | + | 5.17E-76 | DNA ligase | DNA, RNA and nucleotide metabolism |
| PJZEDJXZ_CDS_0026 | 15748 | 15864 | + | No_PHROG | Hypothetical protein | Unknown function |
| PJZEDJXZ_CDS_0027 | 15931 | 16611 | + | 1.92E-28 | Deoxynucleoside monophosphate kinase | Other |
| PJZEDJXZ_CDS_0028 | 16608 | 16766 | + | 3.23E-14 | RNA polymerase inhibitor | DNA, RNA and nucleotide metabolism |
| PJZEDJXZ_CDS_0029 | 16763 | 17134 | + | 2.24E-69 | Hypothetical protein | Unknown function |
| PJZEDJXZ_CDS_0030 | 17208 | 17900 | + | 1.26E-25 | Gp2.5-like ssdna binding protein and ssdna annealing protein | DNA, RNA and nucleotide metabolism |
| PJZEDJXZ_CDS_0031 | 17903 | 18340 | + | 2.61E-69 | Endonuclease | Dna, rna and nucleotide metabolism |
| PJZEDJXZ_CDS_0032 | 18352 | 18813 | + | 1.40E-64 | Amidase | Lysis |
| PJZEDJXZ_CDS_0033 | 18880 | 19416 | + | 2.11E-42 | Nucleotidyltransferase | Dna, rna and nucleotide metabolism |
| PJZEDJXZ_CDS_0034 | 19420 | 21111 | + | 1.41E-79 | DNA primase/helicase | DNA, RNA and nucleotide metabolism |
| PJZEDJXZ_CDS_0035 | 21114 | 21332 | + | 1.04E-30 | Hypothetical protein | Unknown function |
| PJZEDJXZ_CDS_0036 | 21391 | 21831 | + | 6.04E-84 | Hypothetical protein | Unknown function |
| PJZEDJXZ_CDS_0037 | 21818 | 23956 | + | 5.69E-107 | DNA polymerase I | DNA, RNA and nucleotide metabolism |
| PJZEDJXZ_CDS_0038 | 23956 | 24270 | + | 1.32E-19 | Gp5.5-like host HNS inhibition | Moron, auxiliary metabolic gene and host takeover |
| PJZEDJXZ_CDS_0039 | 24273 | 24482 | + | 4.28E-37 | Hypothetical protein | Unknown function |
| PJZEDJXZ_CDS_0040 | 24479 | 25393 | + | 1.57E-40 | Exonuclease | Dna, rna and nucleotide metabolism |
| PJZEDJXZ_CDS_0041 | 26152 | 25472 | - | No_PHROG | Hypothetical protein | Unknown function |
| PJZEDJXZ_CDS_0042 | 26055 | 26372 | + | 1.23E-18 | Host range and adsorption protein | Moron, auxiliary metabolic gene and host takeover |
| PJZEDJXZ_CDS_0043 | 26385 | 27992 | + | 1.65E-177 | Head-tail adaptor | Connector |
| PJZEDJXZ_CDS_0044 | 28058 | 28969 | + | 1.38E-92 | Head assembly | Head and packaging |
| PJZEDJXZ_CDS_0045 | 29058 | 29396 | + | 3.18E-25 | Major head protein | Head and packaging |
| PJZEDJXZ_CDS_0046 | 29326 | 30042 | + | 1.09E-71 | Major head protein | Head and packaging |
| PJZEDJXZ_CDS_0047 | 30084 | 31457 | + | 7.54E-13 | Structural protein with Ig domain | Head and packaging |
| PJZEDJXZ_CDS_0048 | 31524 | 32111 | + | 1.50E-75 | Tail protein | Tail |
| PJZEDJXZ_CDS_0049 | 32122 | 34542 | + | 2.22E-203 | Tail protein | Tail |
| PJZEDJXZ_CDS_0050 | 34572 | 35009 | + | 1.96E-49 | Internal virion protein | Head and packaging |
| PJZEDJXZ_CDS_0051 | 35021 | 35578 | + | 1.85E-46 | Internal virion protein | Head and packaging |
| PJZEDJXZ_CDS_0052 | 35587 | 37782 | + | 9.66E-296 | Internal virion protein | Head and packaging |
| PJZEDJXZ_CDS_0053 | 37804 | 39888 | + | 8.85E-166 | Internal virion protein with endolysin domain | Head and packaging |

**Table S5:** Orthologous protein comparisons between phages vB_PsyP_Mobley and vB_PsyP_Plaza inferred from reciprocal BLASTP best hits.

| **vB_PsyP_Mobley^1^** | **vB_PsyP_Plaza^1^** | **Shared Annotation** | **% Identity** | **E-value** | **Alignment score** |
| --- | --- | --- | --- | --- | --- |
| YCJ12245.1_5 | YCJ12331.1_33 | 2og-fe(ii) oxygenase | 39.13 | 1.12e-56 | 172 |
| YCJ12253.1_13 | YCJ12333.1_35 | Hypothetical protein | 40 | 8.46e-26 | 88.6 |
| YCJ12273.1_33 | YCJ12343.1_45 | Terminase large subunit | 36.283 | 1.39e-92 | 288 |
| YCJ12277.1_37 | YCJ12348.1_50 | Tail fiber protein | 33.871 | 1.17e-07 | 45.1 |
| YCJ12281.1_41 | YCJ12353.1_55 | Tail protein | 24.906 | 2.82e-19 | 83.2 |
| YCJ12282.1_42 | YCJ12300.1_2 | Tail protein | 25.134 | 2.67e-09 | 45.8 |
| YCJ12285.1_45 | YCJ12304.1_6 | Head-tail adaptor | 29.091 | 8.02e-52 | 176 |
| YCJ12289.1_49 | YCJ12328.1_30 | RNA polymerase | 27.986 | 2.47e-91 | 298 |

^1^ Locus tags were obtained from the genome sequences of phages vB_PsyP_Mobley (PX694326) and vB_PsyP_Plaza (PX694327) deposited in NCBI.

**Table S6:** Orthologous protein comparisons between phages vB_PsyP_Mission and vB_PsyP_Mobley inferred from reciprocal BLASTP best hits.

| **vB_PsyP_Mission^1^** | **vB_PsyP_Mobley^1^** | **Shared Annotation** | **% Identity** | **E-value** | **Alignment score** |
| --- | --- | --- | --- | --- | --- |
| YCJ12359.1_6 | YCJ12273.1_33 | Terminase large subunit | 36.59 | 1.28E-90 | 283 |
| YCJ12366.1_13 | YCJ12253.1_13 | Hypothetical protein | 40.517 | 2.04E-27 | 92.8 |
| YCJ12372.1_19 | YCJ12245.1_5 | 2OG-Fe(II) oxygenase | 38.793 | 1.19E-52 | 162 |
| YCJ12374.1_21 | YCJ12289.1_49 | RNA polymerase | 30.552 | 3.12E-99 | 318 |
| YCJ12377.1_24 | YCJ12290.1_50 | ATP-dependent DNA ligase | 24.062 | 2.35E-07 | 42.4 |
| YCJ12390.1_37 | YCJ12241.1_1 | DNA polymerase | 26.54 | 1.67E-07 | 44.7 |
| YCJ12396.1_43 | YCJ12285.1_45 | Head-tail adaptor | 30.181 | 5.16E-55 | 184 |
| YCJ12401.1_48 | YCJ12282.1_42 | Tail protein | 28.261 | 2.78E-08 | 43.1 |
| YCJ12402.1_49 | YCJ12281.1_41 | Tail protein | 22.642 | 4.70E-20 | 85.9 |

^1^ Locus tags were obtained from the genome sequences of phages vB_PsyP_ Mission (PX694328) and vB_PsyP_ Mobley (PX694326) deposited in NCBI.

**Table S7:** Orthologous protein comparisons between phages vB_PsyP_Mission and vB_PsyP_Plaza inferred from reciprocal BLASTP best hits.

| **vB_PsyP_Mission^1^** | **vB_PsyP_Plaza^1^** | **Shared Annotation** | **% Identity** | **E-value** | **Alignment score** |
| --- | --- | --- | --- | --- | --- |
| YCJ12354.1_1 | YCJ12349.1_51 | Internal virion protein | 52.201 | 0 | 654 |
| YCJ12355.1_2 | YCJ12348.1_50 | Tail fiber protein | 41.58 | 7.97E-84 | 266 |
| YCJ12356.1_3 | YCJ12346.1_48 | Holin | 38.71 | 2.60E-10 | 42.7 |
| YCJ12357.1_4 | YCJ12345.1_47 | Terminase small subunit | 54.878 | 6.06E-27 | 86.3 |
| YCJ12358.1_5 | YCJ12344.1_46 | Rz-like spanin | 34.146 | 6.78E-15 | 59.3 |
| YCJ12359.1_6 | YCJ12343.1_45 | Terminase large subunit | 75.26 | 0 | 908 |
| YCJ12360.1_7 | YCJ12341.1_43 | Hypothetical protein | 45.455 | 1.47E-09 | 40 |
| YCJ12366.1_13 | YCJ12333.1_35 | Hypothetical protein | 53.548 | 3.96E-59 | 173 |
| YCJ12367.1_14 | YCJ12335.1_37 | Hypothetical protein | 35.484 | 3.13E-06 | 32.3 |
| YCJ12371.1_18 | YCJ12332.1_34 | Hypothetical protein | 29.193 | 2.05E-13 | 56.6 |
| YCJ12372.1_19 | YCJ12331.1_33 | 2OG-Fe(II) oxygenase | 45.259 | 3.35E-72 | 212 |
| YCJ12374.1_21 | YCJ12328.1_30 | RNA polymerase | 63.507 | 0 | 1078 |
| YCJ12377.1_24 | YCJ12324.1_26 | DNA ligase | 42.776 | 4.97E-93 | 273 |
| YCJ12380.1_27 | YCJ12322.1_24 | Deoxynucleoside monophosphate kinase | 46.429 | 1.09E-59 | 179 |
| YCJ12381.1_28 | YCJ12321.1_23 | RNA polymerase inhibitor | 55.102 | 2.67E-14 | 52 |
| YCJ12382.1_29 | YCJ12320.1_22 | Hypothetical protein | 59.504 | 5.35E-47 | 139 |
| YCJ12383.1_30 | YCJ12319.1_21 | Ssdna binding and annealing protein | 47.436 | 1.68E-63 | 189 |
| YCJ12384.1_31 | YCJ12318.1_20 | Endonuclease | 61.538 | 1.71E-68 | 196 |
| YCJ12385.1_32 | YCJ12317.1_19 | Amidase | 63.265 | 7.31E-67 | 192 |
| YCJ12386.1_33 | YCJ12316.1_18 | Nucleotidyltransferase | 37.714 | 1.46E-33 | 109 |
| YCJ12387.1_34 | YCJ12315.1_17 | DNA primase/helicase | 69.069 | 0 | 804 |
| YCJ12388.1_35 | YCJ12314.1_16 | Hypothetical protein | 41.27 | 2.52E-06 | 32.7 |
| YCJ12389.1_36 | YCJ12313.1_15 | Hypothetical protein | 26.897 | 4.68E-06 | 35.4 |
| YCJ12390.1_37 | YCJ12312.1_14 | DNA polymerase I | 65.928 | 0 | 957 |
| YCJ12391.1_38 | YCJ12311.1_13 | Host HNS inhibition protein | 43 | 1.49E-21 | 74.3 |
| YCJ12392.1_39 | YCJ12310.1_12 | Hypothetical protein | 59.42 | 2.55E-29 | 90.9 |
| YCJ12393.1_40 | YCJ12309.1_11 | Exonuclease | 57.329 | 1.00E-129 | 363 |
| YCJ12395.1_42 | YCJ12305.1_7 | Host range and adsorption protein | 54.369 | 1.03E-11 | 48.5 |
| YCJ12396.1_43 | YCJ12304.1_6 | Head-tail adaptor | 77.528 | 0 | 867 |
| YCJ12397.1_44 | YCJ12303.1_5 | Head assembly protein | 50 | 1.43E-94 | 273 |
| YCJ12399.1_46 | YCJ12301.1_3 | Major head protein | 77.778 | 8.34E-138 | 380 |
| YCJ12401.1_48 | YCJ12300.1_2 | Tail protein | 71.282 | 2.75E-106 | 295 |
| YCJ12402.1_49 | YCJ12353.1_55 | Tail protein | 64.048 | 0 | 850 |
| YCJ12402.1_49 | YCJ12299.1_1 | Tail protein | 75 | 2.19E-75 | 233 |
| YCJ12403.1_50 | YCJ12352.1_54 | Internal virion protein | 46.207 | 1.79E-35 | 112 |
| YCJ12404.1_51 | YCJ12351.1_53 | Internal virion protein | 50.543 | 5.08E-56 | 167 |
| YCJ12405.1_52 | YCJ12350.1_52 | Internal virion protein | 43.388 | 0 | 605 |
| YCJ12406.1_53 | YCJ12349.1_51 | Internal virion protein | 42.167 | 2.22E-171 | 519 |

^1^ Locus tags were obtained from the genome sequences of phages vB_PsyP_ Mission (PX694328) and vB_PsyP_ Plaza (PX694327) deposited in NCBI.
