# Supplementary material for: Genomic and biological characterization of lytic phages infecting Pseudomonas syringae associated with almond bacterial blast
Source: Sci Rep. 2026 Apr 7;16:11657. doi: 10.1038/s41598-026-47496-5 (PMC13061930; doi:10.1038/s41598-026-47496-5)
Supplement: Supplementary file 4 — Supplementary Material 4 [file 41598_2026_47496_MOESM4_ESM.docx]

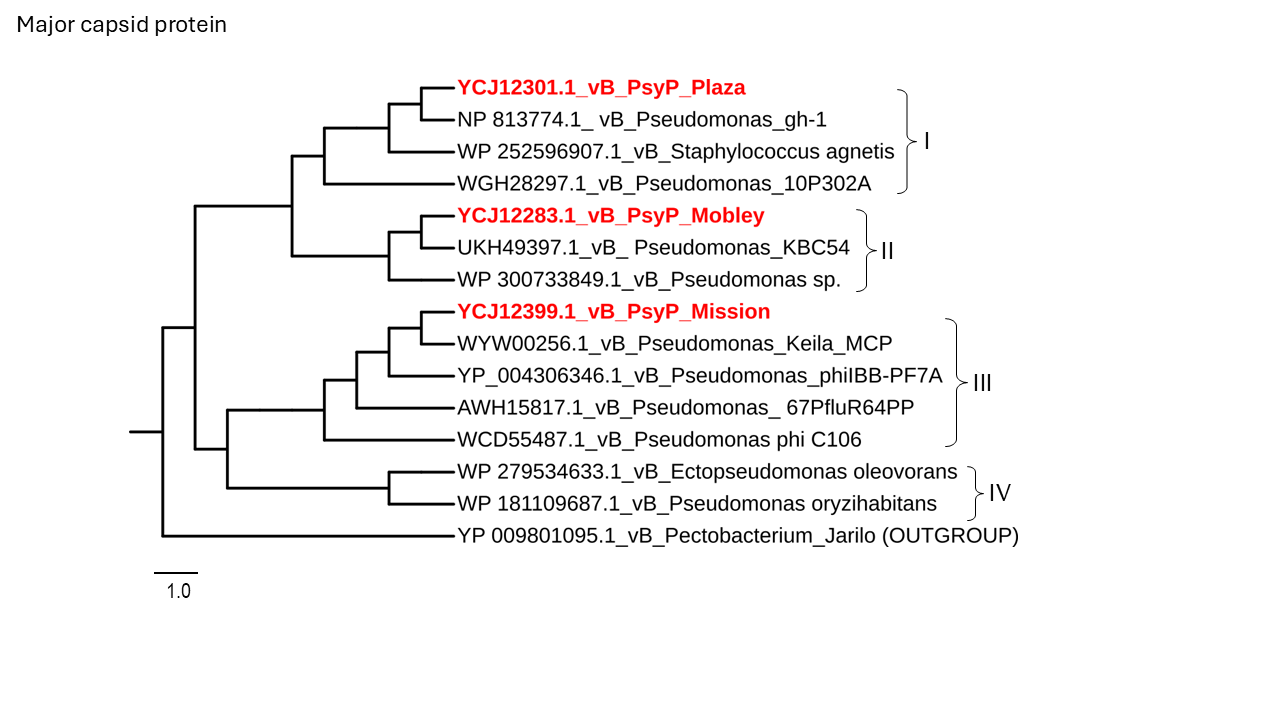


**Figure S1. Major capsid protein (MCP) phylogeny of vB_PsyP phages and close relatives.** Maximum-likelihood tree inferred from MCP amino acid sequences. The three study phages are highlighted in red. Brackets denote the major clades (Groups I–IV) resolved in this analysis. vB_PsyP_Plaza clusters within Group I with its closest relative, Pst_gh1/Pst_GIL1, vB_PsyP_Mobley clusters within Group II with Pseudomonas phage KBC54, and vB_PsyP_Mission clusters within Group III with vB_PpuP-Keila and related phages. Pectobacterium phage Jarilo was used as the outgroup.


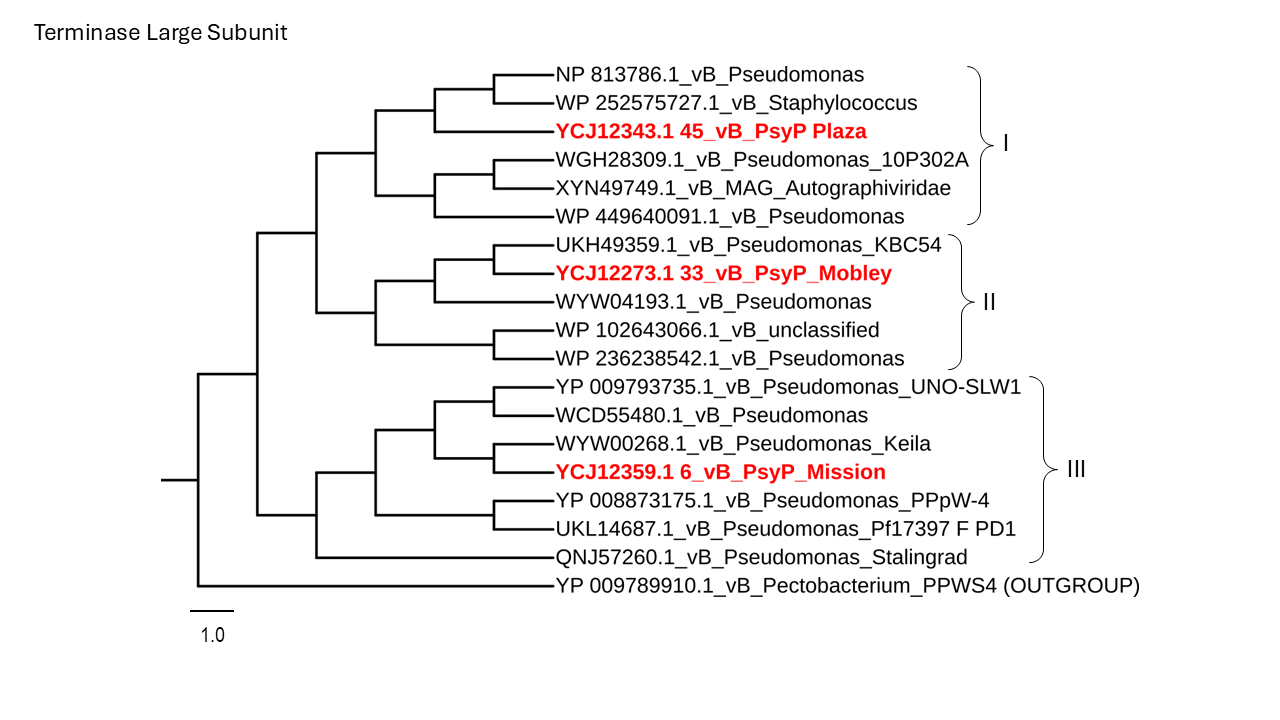


**Figure S2. Terminase large subunit (TerL) phylogeny of vB_PsyP phages and close relatives.** Maximum-likelihood tree inferred from TerL amino acid sequences. The three study phages are highlighted in red. Brackets indicate the major clades recovered in this analysis (Groups I–III). Consistent with the MCP phylogeny, vB_PsyP_Plaza groups with Pst_gh1/Pst_GIL1 (Group I), vB_PsyP_Mobley groups with KBC54 (Group II), and vB_PsyP_Mission groups with vB_PpuP-Keila and related phages (Group III). Pectobacterium phage PPWS4 was used as the outgroup.


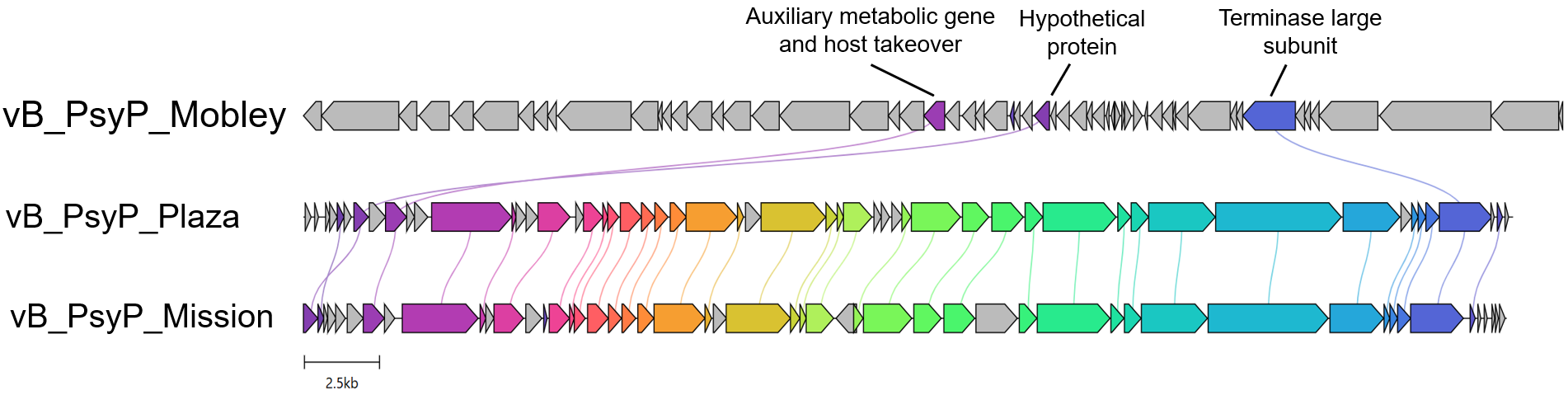


**Figure S3. Comparative genome alignment of vB_PsyP_Mobley, vB_PsyP_Plaza, and vB_PsyP_Mission.** Linear genome comparison showing gene order and shared regions among the three phages. Open reading frames are shown as arrows and colored by functional class. Shaded connectors indicate homologous regions between genomes. Scale bar is 2.5 kb.
